# Validation of an insertion-engineered isoprene synthase as a strategy to functionalize terpene synthases†

**DOI:** 10.1039/d1ra05710c

**Published:** 2021-09-08

**Authors:** C. Raul Gonzalez-Esquer, Bryan Ferlez, Sarathi M. Weraduwage, Henning Kirst, Alexandra T. Lantz, Aiko Turmo, Thomas D. Sharkey, Cheryl A. Kerfeld

**Affiliations:** MSU-DOE Plant Research Laboratory, Michigan State University East Lansing MI 48824 USA ckerfeld@lbl.gov; Department of Biochemistry & Molecular Biology, Michigan State University East Lansing MI 48824 USA; Environmental Genomics and Systems Biology and Molecular Biophysics and Integrated Bioimaging Divisions, Lawrence Berkeley National Laboratory 1 Cyclotron Road Berkeley CA 94720 USA; Plant Resilience Institute, Michigan State University East Lansing MI 48824 USA

## Abstract

Terpene synthases are biotechnologically-relevant enzymes with a variety of applications. However, they are typically poor catalysts and have been difficult to engineer. Structurally, most terpene synthases share two conserved domains (α- and β-domains). Some also contain a third domain containing a second active site (γ-domain). Based on the three-domain architecture, we hypothesized that αβ terpene synthases could be engineered by insertion of a heterologous domain at the site of the γ-domain (an approach we term “Insertion-engineering terpene synthase”; Ie-TS). We demonstrate that by mimicking the domain architecture of αβγ terpene synthases, we can redesign isoprene synthase (ISPS), an αβ terpene synthase, while preserving enzymatic activity. Insertion of GFP or a SpyCatcher domain within ISPS introduced new functionality while maintaining or increasing catalytic turnover. This insertion-engineering approach establishes that the γ-domain position is accessible for incorporation of additional sequence features and enables the rational engineering of terpene synthases for biotechnology.

## Abstract 2

“Insertion-engineering” approach allows for the modification of αβ terpene synthases.

## Graphical abstract

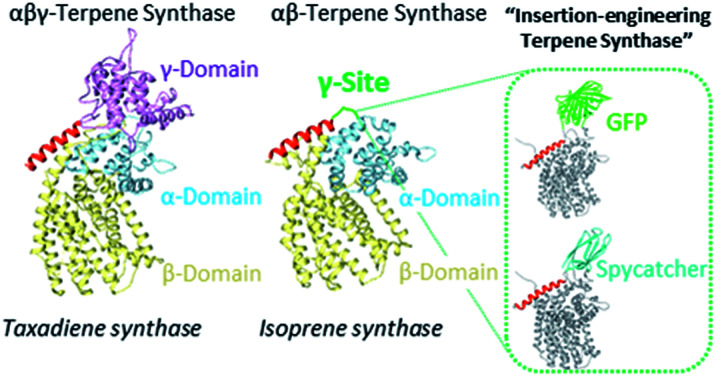

## Introduction

**Fig. 1:**
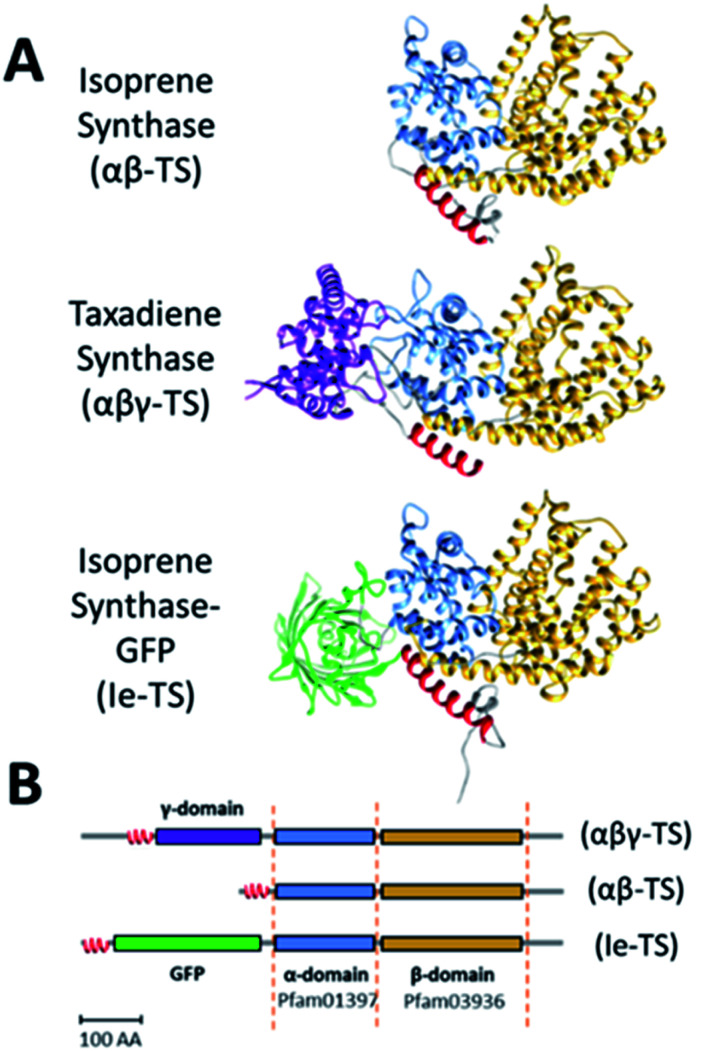
Structural comparison of native and insert-engineered terpene synthases (Ie-TS). (A) Domain structure and (B) domain arrangement of isoprene synthase (αβ-domains), taxadiene synthase (αβγ-domains) and insert-engineered isoprene synthase. The red α-helix is normally part of the α-domain and marks the location of the γ-site. α-Domain: blue; β-domain: yellow; γ-domain: purple; GFP: green.

Terpene synthases are a class of enzymes with potential use in the therapeutic and renewable chemical industries. While about half of terpene synthases produce a single product, others can produce 50 or more products in different reaction cycles.^1^ The array of terpenoid molecules originate from the combination of two metabolic intermediates: dimethylallyl diphosphate (DMADP) and isopentenyl diphosphate (IDP).^2^ Generally, terpene synthases catalyse the condensation of IDP with DMADP or other isoprenoid diphosphate, in a head-to-tail manner by ionizing the diphosphate ester bond to generate a carbocation of the latter substrate, followed by its coupling with the C3=C4 double bond of IDP, and a final deprotonation.^3,4^ Based on the mechanism utilized for carbocation formation, these enzymes are categorized into two main classes, class I and II.^3^ Most terpene synthases contain one α- (Pfam03936) and one β- (Pfam01397) domain (Fig. 1A and B).^3^ The αβ structure is conserved across the variety of biosynthetic functions, as it facilitates the carbocation reaction occurring during the production of the hydrocarbon backbone precursors.^3^ A subset of terpene synthases also contain an additional domain, the γ-domain (no assigned Pfam), that is responsible for the cyclization of the terpenoid backbone.^3^ The αβ and αβγ domain types are the predominant terpene synthases found in plants.^3,5^ In contrast, most fungal and bacterial terpene synthases are single α domain enzymes (*e.g.*, trichodiene synthase, pentalene synthase), but tandem α-α domain (labdane-related diterpene cyclase) and βγ domain (*e.g.*, ent-copalyl diphosphate synthase) are also found in *Streptomyces*.^3,4,6,7^ The αβ domain architecture is only found in class I terpene synthases whereas βγ is only found in class II. The other types of domain architecture are found in all classes. Plant terpene synthases are further categorized into seven clades: TPSa-h (class I: TPS-a, TPS-b, TPS-d, TPS-e/f, TPS-g, TPS-h; class II: TPS-c) based on phylogenetic analyses.^4,8^ The C-terminus α-domain of class I terpene synthases carries an aspartate-rich DDxxD motif. A ‘DxDD’ motif is present in the β-domain of class II terpene synthases. Both motifs are highly conserved and are responsible for catalytic activity.

Because of their diversity and abundance, terpene synthases have myriad potential uses in the specialty chemical industry.^9^ However, terpene synthases are often inefficient industrial catalysts for several reasons: (i) they commonly have a low *k*_cat_ and a high *K*_M_, (ii) they may be inhibited by their substrate, excess Mg^2+^ or Mn^2+^ ions, and (iii) their activity and/or solubility is altered after the modification of the N- or C-terminus.^10–12^ While modifications of terpene synthase active sites result in different product specificity,^13,14^ substantial improvement of the enzyme activity and reduction of substrate inhibition in isoprene synthase (ISPS) by individual amino acid substitutions has been difficult to achieve.^15^ The negative effects incurred on activity upon modifications to the N- or C- terminus have been especially problematic. For example, deletion of up to 79, amino acids from the N-terminus of taxadiene synthase (class I, αβγ domain TPS-d diterpene synthase) resulted in lower activity; deletion of 93 or more residues from the N-terminus completely inactivated the enzyme.^3,5,16^ Similar decreases in activity were seen in N-terminus truncated geraniol synthase.^17^ Truncation or substitution at the N-terminus inactivated limonene synthase, a class I αβ domain TPS-b monoterpene synthase.^18^ Above reductions in activity were attributed to the disruption of an N-terminal domain RR(X)_8_W motif, carrying a conserved pair of tandem arginines and a strictly conserved tryptophan residue.^3,5,16–18^

Isoprene synthase, a class I, TPS-b terpene synthase, catalyses the production of 2-methyl-1,3-butadiene (widely known as isoprene), and is found in many plants, especially trees. It is a model for studying terpene synthase structure and function, as it consists solely of the αβ domains that are structurally conserved throughout most terpene synthases. While isoprene is biologically produced in large amounts in the environment (400–600 Tg C per year), commercially it is produced as a byproduct of petroleum processing.^19^ Engineered isoprene biosynthesis has been achieved in yeast,^20^ cyanobacteria,^21^ and *E. coli*,^22^ among others. Efforts to increase isoprene yields have focused on engineering of precursor pathways,^10,23,24^ decreasing feedback inhibition in relevant enzymes^25^ and improving ISPS expression.^26^ Direct engineering of the ISPS enzyme has focused on N- or C-terminal extensions or truncations or mutation of one or more amino acids in the existing structure that usually resulted in reduced activity of the enzyme.^12,26^ For example, direct linkage of a β-subunit of phycocyanin to the N-terminal catalytic domain of cyanobacterial ISPS led to a reduction in ISPS activity.^26^ Also, the addition of a N- or C-terminal His-tag significantly altered catalytic properties of recombinant poplar ISPS.^12^

To overcome the inhibition caused by N- or C-terminal modifications, we hypothesized that the αβ domain architecture could be modified by inserting a heterologous domain in the position generally occupied by the γ domain in αβγ terpene synthases. This would allow adding new functions to ISPS while avoiding deleterious effects associated with N- or C-terminal modifications. Here, we demonstrate that we can engineer the γ site in the model αβ terpene synthase ISPS without disrupting its catalytic activity. These results suggest recapitulating the modular architecture of γ terpene synthases (an approach we have termed “insertion-engineering terpene synthase”) is a broadly applicable strategy for the functionalization of αβ terpene synthases to support the goals of a sustainable biobased economy.

## Methods

### Structural analysis of terpene synthases and γ-site design

Structures for ISPS (PDB ID: 3N0F)^27^ and taxadiene synthase (PDB ID: 3P5P)^5^ were superimposed using the best-aligned pair of chains using the Matchmaker function of the Chimera software package^28^ for the identification of the γ-site in ISPS (corresponding loop where the γ-domain is found on taxadiene synthase). The *ISPS* gene was modified by introducing a heterologous coding sequence, plus corresponding flanking regions coding for 5× GS linkers, after amino acid 47 of the native ISPS protein, followed by a (4× GS)-RS linker fusing it to the rest of the ISPS enzyme (γ-site). For the generation of the ISPS-GFP model, the sequence of ISPS including the linker was submitted to Swissmodel (https://swissmodel.expasy.org)^29^ which generated a structure based on PDB ID: 3N0F; the structure of GFP (using Swissmodel/PDB ID: 1QYO) was then inserted into the loop manually using Coot^30^ and geometry of the linker was regularized. The combined PDB was then subjected to Rosetta.relax with default parameters.^31^ Chimera^28^ was used for structure visualization and comparison.

### Gene and plasmid design

The *ISPS* sequence from *Populus alba* (Uniprot Accession No. A9Q7C9) was codon optimized for *E. coli* and the chloroplastic targeting peptide was removed from the gene (resulting in gene *ISPS*). All genes were ordered from Genscript Biotech (Piscataway, NJ) and cloned into its corresponding backbone vector *via* Gibson assembly.^32^ ISPS and Ie-ISPS-GFP (Sequences S1 and S2†) variants were assembled into ATUM's pD881 plasmid (Newark, CA) and Ie-ISPS-SpyCatcher variants (Sequences S3–S5†) were assembled into the pBbE2K (from the Bglbrick collection^33^).

### Protein purification

#### Purification of untagged ISPS

*E. coli* BL21 (DE3) cells transformed with pD881/ISPS or pD881/Ie-*ISPS*-GFP plasmids were grown in Erlenmeyer flasks with lysogeny broth (LB) media, in a rotary shaker set at 180 rpm and at 37 °C. Cultures at an OD_600_ of 0.6–0.8 were induced with 3 mM rhamnose, after which cells were further incubated for 16 h at 18 °C. Afterwards, cells were suspended in purification buffer A (50 mM Tris, 10% glycerol) and lysed by two consecutive passage through a French Press at 104 psi in the presence of 1× Sigmafast® protease inhibitor, 0.1 mg ml^−1^ lysozyme and 1 mg ml^−1^ DNase (Sigma Aldrich, St. Louis, MO). The soluble and insoluble fractions of the lysate were separated by centrifugation at 40 000 × *g* for 45 min at 4 °C. The soluble fraction was filtered through a 0.22 μm syringe filter (Milipore Sigma, Burlington, MA). The clarified supernatant was eluted from a HiTrap Q HP 5 ml column (General Electric, Boston, MA) followed by further purification on a HiLoad 26/600 Superdex 200 pg (General Electric, Boston, MA). Protein purity was determined by SDS-PAGE following the manufacturer's protocol (Bio-Rad Bulletin 6201). Protein concentration was determined using the Lowry assay.

#### Purification of SUMO-tagged Ie-ISPS-GFP

The pD881/Ie-ISPS-GFP construct was modified by including an N-terminal cleavable Strep-SUMO-tag to facilitate enzyme purification. A 2 l culture of *E. coli* BL21 (DE3) expressing the Strep-SUMO-tagged proteins were harvested by centrifugation and resuspended in buffer B (50 mM Tris pH 8.0, 200 mM NaCl, 5 mM KCl, 50 mM MgCl_2_, 5% glycerol, 0.02% sodium azide, 0.5 mM phenylmethylsulfonyl fluoride, 1 mM tris(2-carboxyethyl) phosphine) to a density of 0.5 g ml^−1^. The cells were immediately lysed and prepared as described above for protein purification. The resulting samples were loaded on a 5 ml StrepTrap column (General Electric, Boston, MA), washed with buffer B and the protein was eluted using buffer B with 2.5 mM desthiobiotin (Sigma Aldrich, St. Louis, MO). The eluted protein was cleaved overnight with Thermo Fisher HIS-tagged Ubl-specific protease 1 (ULP1) at 4 °C (Waltham, MA). Protein purity and concentration was determined as described above.

### Quantification of isoprene synthase activity

Purified proteins were diluted to 10 pmol of protein per assay. DMADP was obtained from Isoprenoids LC (Tampa, FL) and dissolved in 2 mM ammonium bicarbonate, pH 7.8. The methodology to measure isoprene was modified from Weise *et al.*^34^ Assays were performed by mixing 10 μl protein solution with assay buffer (50 mM HEPES pH 8, 10 mM MgCl_2_, 20 mM KCl, 10% glycerol v/v) and DMADP to a final volume of 100 μl in a 2 ml crimp top glass vial (Supelco, PA). Extracts were then incubated for 15 min in a 40 °C water bath. One ml of headspace was pulled from the vial simultaneously as 1 ml of water was injected (to prevent creating a vacuum). The amount of isoprene in the headspace sample was immediately measured on the Hills-Scientific Fast Isoprene Sensor (FIS) (Boulder, CO) as described in Guenther and Hills.^35^ To obtain the rate of isoprene emission, first the total amount of isoprene in the reaction vial (isoprene in the gas phase + liquid phase) was calculated. The amount of isoprene in the liquid phase was calculated using the pressure of the gas phase, which was assumed to be atmospheric pressure (98 kPa in East Lansing) and the Henry's constant at 40 °C (12 770 247 Pa l mol^−1^).^34^ To determine the rate of isoprene emission, the total amount of isoprene that accumulated in the reaction vial during incubation was divided by the amount of protein used in the assay, and the incubation period (0.25 h).

For measurements where DMADP was held constant, a final concentration of 1 mM DMADP was used as this concentration yielded the highest activity for most extracts. When testing the effect of pH on activity of the engineered ISPS enzymes, assay buffer was made up with MES (pH 6 and 6.5) or HEPES (pH 6.5 to 8.4). Activity of engineered ISPS enzymes under different metal ion concentrations was performed by removing metal ions from the buffer and supplementing accordingly into the assay mixture. For each protein variant, three independent (separate enzyme purifications) trials were performed to determine activity and response to DMADP; each trial had itself three technical replicates at each DMADP concentration. For response to temperature, pH, and ion concentration only one trial was performed. Non-linear curve fitting was performed using a least-squares regression using OriginLab Corporation's Origin 8 (Northampton, MA). DMADP data was fitted to the equation from Reed *et al.*^36^ that allows for substrate inhibition: rate = *k*_cat_/{1 + (*K*_M_/[S]) + ([S]/*K*_I_)}.

### Preparation of cell lysates for isoprene production and SDS-PAGE analyses

Five ml of each starter culture from strains expressing either wild-type ISPS (WT ISPS), Ie-ISPS-SpyCatcher, an N-terminal truncation of Ie-ISPS-SpyCatcher lacking the first 53 amino acids (encoding the N-terminal alpha helix), or a negative control protein lacking SpyCatcher (see ESI Table S1† for full amino acid sequences of all ISPS variants) were used to inoculate 500 ml of lysogeny broth containing 50 μg ml^−1^ kanamycin and grown at 37 °C and 180 rpm. At an OD_600_ of ∼0.6, cultures were induced with 50 ng ml^−1^ anhydrotetracycline (aTc) and shaken at 180 rpm for 21 h at 18 °C. Cultures were collected by centrifugation at 5000 × *g* for 20 min at 4 °C. The resulting cell pellets from 250 ml of each culture were resuspended in a lysis buffer containing lysozyme and Benzonase nuclease (Qproteome Bacterial Protein Prep Kit, Qiagen, MD) and EDTA free protease inhibitor (Sigma-Aldrich, MO) and incubated for 30 min on ice. Cells were lysed using a sonicator (Sonifier® 250 Cell Disrupter, Branson Ultrasonics, VWR, PA) set at a 50% duty cycle. Each lysate sample was subjected to four cycles of 15-ultrasonic pulses with an amplitude output set at 1, followed by two more cycles of 15-ultrasonic pulses (one pulse per s) with an amplitude output set at 2. The horn tip of the sonicator was cooled with ice water for 30 s between cycles. Afterwards, the lysed cell suspension was centrifuged at 4500 × *g* for 20 min at 4 °C to separate the lysate from cell debris.

Total protein concentration of each clarified lysate was determined by the absorbance at 280 nm and corrected for nucleic acid content using the absorbance at 260 nm and the following equation: protein concentration (mg ml^−1^) = (1.55 × *A*_280_) − (0.76 × *A*_260_).^37^ For SDS-PAGE analyses, 4 μg of total protein was loaded per well of a BioRad AnyKD gel. The methodology to measure isoprene was modified from Weise *et al.*^34^ To measure isoprene formation, 5 μl of each clarified cell lysate was added to 295 μl of assay buffer (50 mM HEPES pH 8, 10 mM MgCl_2_, 20 mM KCl, 2 mM DTT, 1 mM EDTA) containing 1 mM DMADP. Reaction mixtures were mixed in a 2 ml crimp top glass vial (Supelco, PA) and isoprene synthesis initiated by adding the cell lysate. The glass vial was then immediately crimp-sealed and incubated for 10 min in a 40 °C water bath. Afterwards, isoprene was measured by a FIS as described above. Independent sample *T*-tests or one-way ANOVA followed by a Fisher test were performed to compare means of isoprene emission rates measured from cell lysates.

### Growth and purification of SpyTag-GFP-His_6_

An overnight culture of *E. coli* BL21 (DE3) cells containing a tet-inducible plasmid encoding SpyTag-GFP-His_6_ (see ESI Table S1† for full amino acid sequence) was used to inoculate 1 l of lysogeny broth containing 50 μg ml^−1^ kanamycin at a dilution of 1 : 100 (v/v). Cells were grown at 37 °C while shaking at 200 rpm until the OD_600_ ∼ 0.6, at which point protein expression was induced by adding aTc to a final concentration of 100 ng ml^−1^. The temperature was then lowered to 18 °C and the culture was shaken for 25 h at 200 rpm. Cells were harvested by centrifugation at 8000 × *g* for 10 min at 4 °C and the resulting cell pellet resuspended in Tris-buffer saline (TBS) solution containing 50 mM Tris–HCl pH 8.0 and 200 mM NaCl (Buffer A) to a final density of 0.5 g ml^−1^. Cells were lysed by two passages through a French Pressure Cell at 2 × 104 psi after incubation on ice for 20 min with 100 μg ml^−1^ lysozyme, 1 mg of DNase, and a SigmaFast protease inhibitor cocktail at 1× final concentration. Unbroken cells and debris were removed by centrifugation at 40000 × *g* for 45 min at 4 °C. Soluble material was filtered through a 0.22 μm syringe filter and loaded at a flow rate of 5 ml min^−1^ onto a 5 ml HisTrap column equilibrated with buffer A using an Akta Pure FPLC system at 10 °C (GE Healthcare). Bound SpyTag-GFP-His_6_ was washed with five column volumes (CV) of Buffer A before eluting with a linear gradient from 0 to 500 mM imidazole in Buffer A over 10 CV at a flow rate of 5 ml min^−1^. Elution fractions were analyzed by SDS-PAGE and those containing pure SpyTag-GFP-His_6_ were pooled and concentrated to a final volume of 2.5 ml using an Amicon 10 kDa MWCO centrifugal filter (Sigma) at 4500 × *g* and 4 °C. Elution buffer was then replaced with Buffer A containing 10% glycerol using a PD-10 desalting column (GE Healthcare). The concentration of purified SpyTag-GFP-His_6_ was determined using an extinction coefficient of 83 300 M^−1^ cm^−1^ at 485 nm.^38^

### Ie-ISPS-SpyCatcher conjugation with SpyTag-GFP-His_6_ in whole cell lysates for enzyme quantification

The same clarified whole cell lysates of strains expressing Ie-ISPS-SpyCatcher (Sequence S3†), or a negative control protein lacking the SpyCatcher domain, used for isoprene production measurements were also used for conjugation reactions with purified SpyTag-GFP-His_6_ (Sequence S4†).

Reactions were carried out in a 15 μl volume containing 10 μg of total protein from each clarified whole cell lysate. Reactions were incubated for 30 min at room temperature with 0, 1, 2, 5, or 10 μM of purified SpyTag-GFP-His_6_ in 50 mM Tris–HCl pH 8.0, 200 mM NaCl, and 10% glycerol. Each conjugation reaction was stopped by addition of 5 μl of SDS-PAGE sample buffer (50 mM Tris–HCl pH 6.8, 2% SDS, 10% glycerol, 1% β-mercaptoethanol, 12.5 mM EDTA, 0.02% bromophenol blue) and boiling for 10 min. Eight μl of each SDS-PAGE sample (equivalent to 4 μg of total protein from the cell lysate) was loaded per well of a commercial Bio-Rad AnyKD acrylamide gel and run at 120 V for 70 min at room temperature; proteins were visualized by staining with a solution of 10% acetic acid, 45% ethanol, and a mixture of Coomassie Blue R-250 (2.4 mM) and G-250 (0.6 mM). Gels were imaged with a Bio-Rad ChemiDoc MP imager (ESI Fig. S1†). Densitometry analyses to determine relative ISPS protein content in *E. coli* cell lysates were performed using ImageJ software (National Institutes of Health and the Laboratory for Optical and Computational Instrumentation, University of Wisconsin), by generating histograms for each lane of the gel and measuring the area under each peak in each histogram. For each lane, the density of the WT and Ie-ISPS variant protein bands were expressed as a percentage of the total density of all protein bands of that lane. Details for the densitometry analysis are described below.

### Densitometry for determining target protein content in cell lysates

#### Wild-type ISPS protein content

WT ISPS (68 kDa) was detected by its migration distance and abundance relative to molecular weight standards on gels loaded with 4 μg total protein from clarified lysate not subjected to conjugation reactions with SpyTag-GFP-His_6_ (ESI Fig. S1†). First, ImageJ software was used to plot histograms for each lane of the gel. The density of each protein band was determined by measuring the area under each peak in each histogram. For each lane, the density of the WT ISPS protein band was expressed as a percentage of the total density of all protein bands of that lane.

#### Ie-ISPS-SpyCatcher content

In contrast to WT ISPS, only a small amount of Ie-ISPS-SpyCatcher (72 kDa) could readily be observed in clarified whole cell lysates using SDS-PAGE (ESI Fig. S1 and S2†) and was obscured by co-migration with an unrelated band at a similar molecular weight. To quantify the amount of Ie-ISPS-SpyCatcher, as well as confirm the function of its SpyCatcher domain, these lysates were subjected to conjugation experiments with SpyTag-GFP-His_6_ prior to SDS-PAGE analysis (see ESI Fig. S2† for the uncropped gel image shown in Fig. 4). The protein band in the gel corresponding to the covalent SpyTag-GFP-His_6_ + Ie-ISPS-SpyCatcher fusion protein (102.2 kDa) was identified by its migration distance relative to protein molecular weight standards as well as the dose-dependence of its intensity as a function of SpyTag-GFP-His_6_ concentration in the conjugation reaction (ESI Fig. S2†). The density of each protein band for each lane on the gel was determined using ImageJ software as described above for WT ISPS. For each lane, the density of the SpyTag-GFP-His_6_ + Ie-ISPS-SpyCatcher fusion protein band was expressed as a percentage of the total density of all protein bands of that lane. The total protein content was adjusted to account for the unconjugated SpyTag-GFP-His_6_ by subtracting the band density of the unconjugated SpyTag-GFP-His_6_ at 29.9 kDa from the total density of all protein bands of the corresponding lane. A minor background protein migrating close to the SpyTag-GFP-His_6_ + Ie-ISPS-SpyCatcher fusion protein could be seen in the histogram corresponding to the lane loaded with the reaction mixture that did not include any SpyTag-GFP-His_6_ (0 μM). Assuming the abundance of this *E. coli* protein is independent of the amount of SpyTag-GFP-His_6_ present, we subtracted the intensity of this peak from the calculated intensity for the SpyTag-GFP-His_6_ + Ie-ISPS-SpyCatcher fusion proteins in the other four reaction mixtures containing 1, 2, 5 and 10 μM SpyTag-GFP-His_6_. The % of the unreacted Ie-ISPS-SpyCatcher (72 kDa) was also determined using the same methodology described above.

### Determination of *k*_cat_ of the Ie-ISPS-SpyCatcher fusion protein and demonstration of the SpyTag-GFP-His_6_ and ISPS-SpyCatcher conjugation reaction

For each conjugation reaction, the amount of SpyTag-GFP-His_6_ + Ie-ISPS-SpyCatcher fusion protein expressed as a % of the SpyTag-GFP-His_6_ + Ie-ISPS-SpyCatcher fusion protein detected with 10 μM of SpyTag-GFP-His_6_ (plot of appearance of SpyTag-GFP-His_6_ + Ie-ISPS-SpyCatcher fusion protein) (ESI Fig. S3†), and the amount of unreacted Ie-ISPS-SpyCatcher expressed as a % of Ie-ISPS-SpyCatcher in the absence of SpyTag-GFP-His_6_ (plot of disappearance of unreacted Ie-ISPS-SpyCatcher), were plotted against the concentration of SpyTag-GFP-His_6_ added to each corresponding conjugation mixture. Plots were fit with a rectangular hyperbola (*Y* = *B*_max_[GFP]/(*K*_d_ + [GFP])) to estimate *B*_max_ (maximum number of binding sites of Ie-ISPS-SpyCatcher) and *K*_d_ (dissociation constant at equilibrium). The amount of Ie-ISPS-SpyCatcher fusion protein in the lysate (determined by densitometry analyses described above) was used to convert the isoprene emission rates expressed per mg of total protein per h to per mg of Ie-ISPS-SpyCatcher protein per h. These isoprene emission rates were used to estimate *k*_cat_ of the fusion protein (*k*_cat_ = *V*_max_/[total enzyme]) considering the enzyme concentration to be 9.8 nmol mg^−1^ based on the molecular weight of the SpyTag-GFP-His_6_ + Ie-ISPS-SpyCatcher fusion protein (102.2 kDa). Statistical analyses were performed as described above.

## Results and discussion

We devised a strategy based on mimicry of the domain architecture of natural αβγ terpene synthases,^3^ to design an insert-engineered terpene synthase (Ie-TS) by inserting a heterologous protein domain internally in the primary structure of the model hemiterpene synthase, ISPS (Ie-ISPS). To introduce a heterologous domain within ISPS, we compared primary and tertiary structure information for the hemiterpene ISPS (αβ domain composition) and the diterpene synthase taxadiene synthase (αβγ domain composition) to demarcate a potential insertion site in the ISPS enzyme (Fig. 1A). This site (hereafter the γ-site) is located between an N-terminal α-helix essential for activity^27^ and the beginning of the α-domain (Fig. 1B); in the *Populus alba* ISPS, this linker region consists of seven amino acids. We selected the 27 kDa GFP protein as an “insertion-engineering domain” for ease of tracking during purification and as a proof-of-concept for tolerance to introducing a foreign domain of average size into the γ-site of *P. alba* ISPS.

**Fig. 2:**
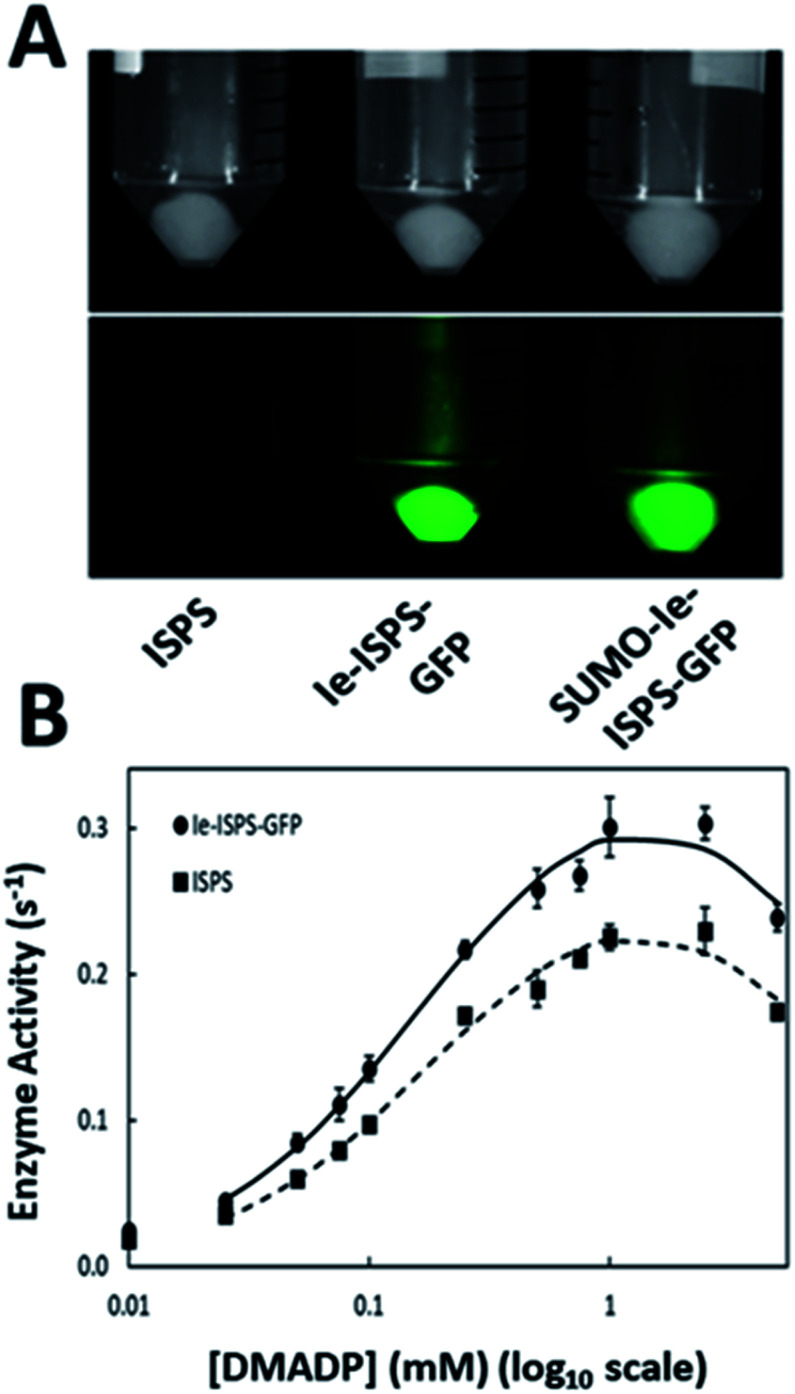
GFP incorporation and enzyme activity of Ie-ISPS-GFP. (A) *E. coli* cells expressing ISPS (left), Ie-ISPS-GFP (middle), or SUMO-Ie-ISPS-GFP (right) imaged without (top) or with (bottom) blue light excitation to show GFP fluorescence. (B) Isoprene synthase activity of Ie-ISPS-GFP (SUMO tag removed) *vs.* ISPS. Averages with standard error shown (*n* = 3). Lines are smoothed and drawn in Excel.

The *P. alba ISPS* sequence lacking its putative chloroplast targeting peptide has been previously expressed and tested in *E. coli*.^11,12,39^ A synthetic gene consisting of the insert-engineered isoprene synthase (Ie-*ISPS*) fusion was cloned into the pD881 vector (Sequence S1†). Preliminary expression testing for this construct demonstrated the proper assembly of GFP, shown by its fluorescence under blue light (Fig. 2A-middle). However, decreased protein solubility was also observed. We addressed this through the addition of a SUMO-tag, previously shown to increase the solubility of difficult-to-express proteins^40,41^ (Sequence S2†). While the SUMO-tag did not affect GFP fluorescence (Fig. 2A-right), it was removed from purified SUMO-Ie-ISPS-GFP using the ULP1 protease (which leaves a scarless cleavage site) prior to comparing its activity to the unmodified WT ISPS to ensure that our assays were conducted in biochemically comparable conditions (Fig. 2B).

**Fig. 3:**
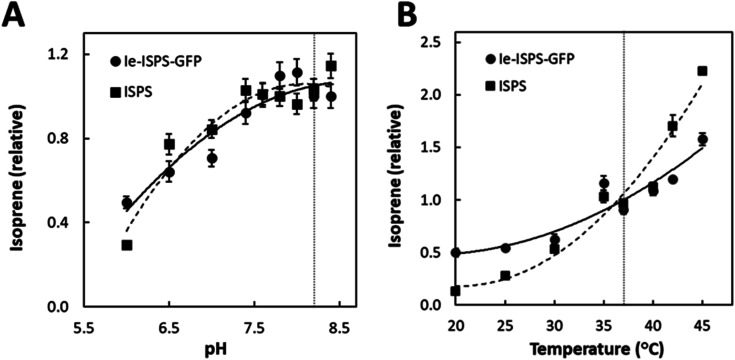
Isoprene evolved by ISPS variants in various pH and temperature conditions. Response of ISPS and Ie-ISPS-GFP to (A) pH (relative to pH 8.2) and (B) temperature (relative to 37 °C). Data were collected with untagged ISPS and Ie-ISPS-GFP. Averages with standard error shown (*n* = 3). The Arrhenius plots were linear consistent with no thermal deactivation and the activation energy for ISPS was −83.7 kJ mol^−1^ while for Ie-ISPS-GFP it was −35.6 kJ mol^−1^. Lines are second-order polynomials.

The enzyme kinetics of purified ISPS and Ie-ISPS-GFP are shown in Table 1. The *K*_M_ and *k*_cat_ of the chimeric enzyme Ie-ISPS-GFP were comparable to the unmodified enzyme. We also compared the effect of pH and temperature on the enzymatic production of isoprene since these are known to vary with N- and C-terminal additions to ISPS.^12^ The Ie-ISPS-GFP enzyme had approximately the same pH response as ISPS (Fig. 3A). The temperature response was less for the Ie-ISPS-GFP than for ISPS (Fig. 3B). The inhibitory effects of the ions Na^+^ and K^+^, and dependence on either Mn^2+^ or Mg^2+^ on Ie-ISPS-GFP were approximately the same as for ISPS (ESI Fig. S4†).

**Table 1:** Kinetic parameters of purified ISPS and Ie-ISPS-GFP. Data of Fig. 2B fitted with an enzyme kinetics equation allowing for substrate inhibition. The averaged data were fitted because, with strong substrate inhibition, the noise in each curve allowed a variety of fittings (many local minima found in the non-linear Solver fittings). The averaged data converged on a robust fitting. The fitting was based on 65 measured rates of isoprene emission from six separate bacterial extracts

	ISPS	Ie-ISPS-GFP
*K*_M_ (mM)	0.19	0.18
*k*_cat_ (s^−1^)	0.29	0.37
*K*_I_ (mM)	9.03	11.12

The insertion of GFP at the γ-site proves that ISPS (and likely other αβ TS) can be engineered from within as opposed to the enzyme's termini, while conserving its form and function. This may reverse the evolutionary step of γ domain loss.^42^

We examined whether or not ISPS could accommodate installation of a functional scaffolding domain using the Ie-TS approach. For this purpose, we generated an Ie-ISPS variant harboring a SpyCatcher domain^43^ in the γ-site (ESI Table S1†). SpyCatcher is an engineered 83 amino acid domain that can covalently bind a target displaying its cognate 13 amino acid SpyTag peptide, enabling the precise control of protein–protein interactions for metabolic optimization *in vivo*. To determine the relative activity of the Ie-ISPS-SpyCatcher design, we measured isoprene formation in clarified *E. coli* lysates from strains expressing either WT ISPS or Ie-ISPS-SpyCatcher with added DMADP. Lysates containing Ie-ISPS-SpyCatcher produced 1.32 ± 0.39 μmol_isoprene_ per mg total protein per h, roughly 18% of the 7.44 ± 1.41 μmol_isoprene_ per mg total protein per h formed by WT ISPS (ESI Fig. S5A†). In contrast, lysates from strains expressing either a truncated form of Ie-ISPS-SpyCatcher lacking the N-terminal alpha helix preceding the γ-site (ΔNterm Ie-ISPS-SpyCatcher, Sequence S5†), or a negative control protein expressed from the same vector as all our ISPS variants, produced only 0.012 ± 0.002 and 0.005 ± 0.0003 μmol_isoprene_ per mg total protein per h, respectively (ESI Fig. S5A†). These results suggest that Ie-ISPS-SpyCatcher is active and that the alpha helix containing a double arginine at the N-terminus of Ie-ISPS-SpyCatcher is required for folding and/or catalysis, as previously reported by Williams *et al.*^18^ and Chaves *et al.*^26^

Next, we estimated the enzyme content of each cell lysate. Purification of ISPS can cause it to lose activity and tags on either the N- or C-terminus to aid purification can alter the kinetic characteristics.^12^ Therefore, we used SDS-PAGE and densitometry analyses to assess the amount of soluble enzyme present in the cell lysates.

**Fig. 4:**
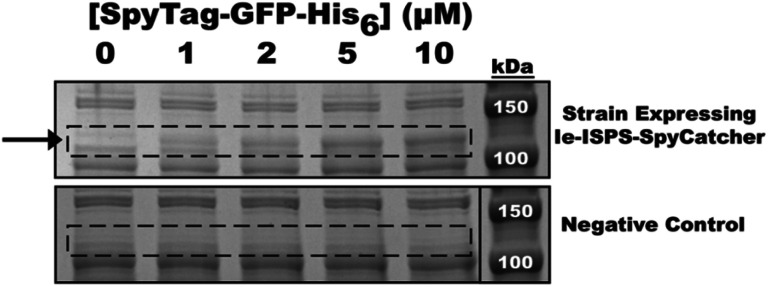
Conjugation of SpyTag-GFP-His_6_ to ISPS-SpyCatcher in clarified whole cell lysates. Coomassie-stained SDS-PAGE gel of conjugation reactions with purified SpyTag-GFP-His_6_ and clarified whole cell lysates from strains expressing either Ie-ISPS-SpyCatcher (top) or a negative control protein lacking the SpyCatcher domain (bottom). As the concentration of SpyTag-GFP-His_6_ is increased from 0 to 10 μM, a protein (marked by an arrow) consistent with the size of the Ie-ISPS-SpyCatcher + SpyTag-GFP-His_6_ covalent fusion (∼102.2 kDa) accumulates in a dose-dependent manner in lysates from the strain expressing Ie-ISPS-SpyCatcher but not in the negative control strain. The molecular weight marker in the bottom panel is the same as the one shown in the top (see Fig. S2† for the uncropped image).

For lysates containing WT ISPS, an intense band was observed with a molecular weight consistent with the full-length enzyme (68 kDa) representing 20% of the total protein content (ESI Fig. S1†). In contrast, only a minor band consistent with the size of Ie-ISPS-SpyCatcher (72 kDa) could be identified in its corresponding lysate. Moreover, a contaminating band with a similar molecular weight as Ie-ISPS-SpyCatcher was also observed in lysates expressing the negative control protein. The low abundance of Ie-ISPS-SpyCatcher and co-migration with a background *E. coli* protein complicated densitometry analysis. To overcome this, and to demonstrate the function of the SpyTag/SpyCatcher system in the Ie-ISPS, SpyTag-GFP-His_6_ was conjugated to Ie-ISPS-SpyCatcher and the resulting band at 102.2 kDa was used to quantify Ie-ISPS-SpyCatcher content. To do this, cell lysates were incubated with 0, 1, 2, 5, or 10 μM of purified SpyTag-GFP-His_6_ for 30 min, SDS-PAGE samples were then prepared and equivalent amounts of total lysate protein (not including the added SpyTag-GFP-His_6_) were run on an SDS-PAGE gel. As we increased the amount of SpyTag-GFP-His_6_ added to lysates of Ie-ISPS-SpyCatcher, the intensity of a new protein band consistent with the molecular weight of the SpyTag-GFP-His_6_ + Ie-ISPS-SpyCatcher fusion (102.2 kDa) increased (Fig. 4; ESI Fig. S2 and S3†).

As a control, SpyTag-GFP-His_6_ was also added to lysates from our negative control strain which encodes a reference protein lacking the SpyCatcher domain; no change in the intensity of bands around 100 kDa was observed in these control reactions. Furthermore, concomitant with the increase in intensity of the SpyTag-GFP-His_6_ + Ie-ISPS-SpyCatcher fusion protein band at 102.2 kDa, we also observed a proportional decrease in the intensity of the faint band assigned to unconjugated Ie-ISPS-SpyCatcher (72 kDa). By fitting a rectangular hyperbola to the data for the appearance of the SpyTag-GFP-His_6_ + Ie-ISPS-SpyCatcher fusion protein, we estimated that the maximum amount of fusion protein was 134% of the highest value we measured and the dissociation constant was 3.45 μM SpyTag-GFP-His_6_ (ESI Fig. S3†). Based on densitometry analysis, the average protein content of SpyTag-GFP-His_6_ + Ie-ISPS-SpyCatcher at 10 μM SpyTag-GFP-His_6_ was 2% of total protein content. The maximum possible amount of fusion protein was estimated to be 1.34 times more than the maximum amount of the SpyTag-GFP-His_6_ + Ie-ISPS-SpyCatcher fusion initially determined by densitometry. Therefore, the corrected average protein content of Ie-ISPS-SpyCatcher was calculated to be 2.72% of total protein content. In contrast, WT ISPS was 20.4% of total protein content. After taking into account these estimated protein amounts, enzyme activities relative to protein mass estimated for Ie-ISPS-SpyCatcher was slightly higher than WT ISPS (ESI Fig. S5B†).

After converting protein mass to moles, the estimated *k*_cat_ of WT ISPS was 0.69 ± 0.07 s^−1^ and for Ie-ISPS-SpyCatcher it was 1.37 ± 0.01 s^−1^ (*n* = 3). The means were statistically different at *α* = 0.05. The variation in the *k*_cat_ in WT ISPS presented here and in Table 1 may reflect the use of purified enzymes for Table 1, but cell lysates for the conjugation experiment. The estimated *k*_cat_ for Ie-ISPS-SpyCatcher falls in the high end of the range of *k*_cat_ values for ISPS. A *k*_cat_ of 0.26 s^−1^ was reported for purified non-recombinant wild-type ISPS from *Populus x canescens* (grey poplar) ISPS.^8^ The highest reported ISPS *k*_cat_ of 4.4 s^−1^ for purified recombinant ISPS from *Pueraria montana* (Kudzu)^6^ was ascribed to removal of the hexahistidine tag, careful choice of N-terminal truncation, and careful removal of inhibitory Ni^+^ ions. While the observed activity of the Ie-ISPS-SpyCatcher fusion protein further validates the Ie-TS strategy, its interaction with the cognate SpyTag domain fused to GFP also demonstrates a practical approach for the utilization of the γ-site for pre-designed domain interactions, for example, in enzyme scaffolds to facilitate substrate channelling, cellular location, and enzyme stabilization.

## Conclusion

Here, we demonstrate a new strategy (the Ie-TS approach) to modify ISPS, a model bi-domain terpene synthase. Rational engineering of terpene synthases is a major objective in bioengineering. We expect the Ie-TS approach to be applicable to even evolutionarily distant bidomain terpene synthases. This architectural template approach may enable, for example, the precise control of protein–protein interactions *in vivo*, or the engineering of new and/or improved catalytic functions.

## Author contributions

CRGE, BF, HK, CAK and TDS designed experiments. BF, SW, ATL, AT carried out the experiments. CRGE, BF, SW, CAK and TSD performed data analysis and prepared the manuscript. All authors read and approved the final manuscript.

## Conflicts of interest

The author's state that there are no conflicts to declare.

## Supplementary Material

RA-011-D1RA05710C-s001
