# Supplementary material for: Validation of an insertion-engineered isoprene synthase as a strategy to functionalize terpene synthases
Source: RSC Adv. 2021 Sep 8;11(48):29997–30005. doi: 10.1039/d1ra05710c (PMC9041124; doi:10.1039/d1ra05710c)
Supplement: RA-011-D1RA05710C-s001 [file RA-011-D1RA05710C-s001.pdf]

Electronic Supplementary Material (ESI) for RSC Advances.
This journal is © The Royal Society of Chemistry 2021

Supplementary Material

Validation of an insertion-engineered isoprene synthase as a strategy to functionalize terpene synthases

C. Raul Gonzalez-Esquer,†[a,b] Bryan Ferlez,†[b,c] Sarathi M. Weraduwage,†[b,c] Henning Kirst,[b,d] Alexandra T. Lantz,[b,c,e] Aiko Turmo,[b,c] Thomas D. Sharkey[b,c,f] and Cheryl A. Kerfeld*[b,c,d]

[a]Bioscience Division, Los Alamos National Laboratory, P.O. Box 1663, MS888, Los Alamos, NM 87545 USA (current); [b]MSU-DOE Plant Research Laboratory, Michigan State University, East Lansing, MI 48824 USA; [c]Department of Biochemistry & Molecular Biology, Michigan State University, East Lansing, MI 48824 USA; [d]Environmental Genomics and Systems Biology and Molecular Biophysics and Integrated Bioimaging Divisions, Lawrence Berkeley National Laboratory, 1 Cyclotron Road, Berkeley, CA 94720 USA; [e]KBI Biopharma, Durham, NC 27704 USA (current); [f]Plant Resilience Institute, Michigan State University, East Lansing, MI 48824 USA.

† Co-first authors; * Corresponding author.

Figure S1: ISPS abundance in clarified *E. coli* cell lysates
Figure S2: Uncropped gel image of SpyTag-GFP-His_6 conjugation reactions
Figure S3: The plot of appearance of SpyTag-GFP-His_6 + Ie-ISPS-SpyCatcher fusion protein
Figure S4: Response of Ie-ISPS-GFP to Na^+ and K^+ concentrations, and Mn^{2+} and Mg^{2+}
Figure S5: Isoprene synthase activity of Ie-ISPS-SpyCatcher
Table S1: Amino acid sequences for proteins used in this study;
Sequences S1-5: Plasmids sequences used in this study

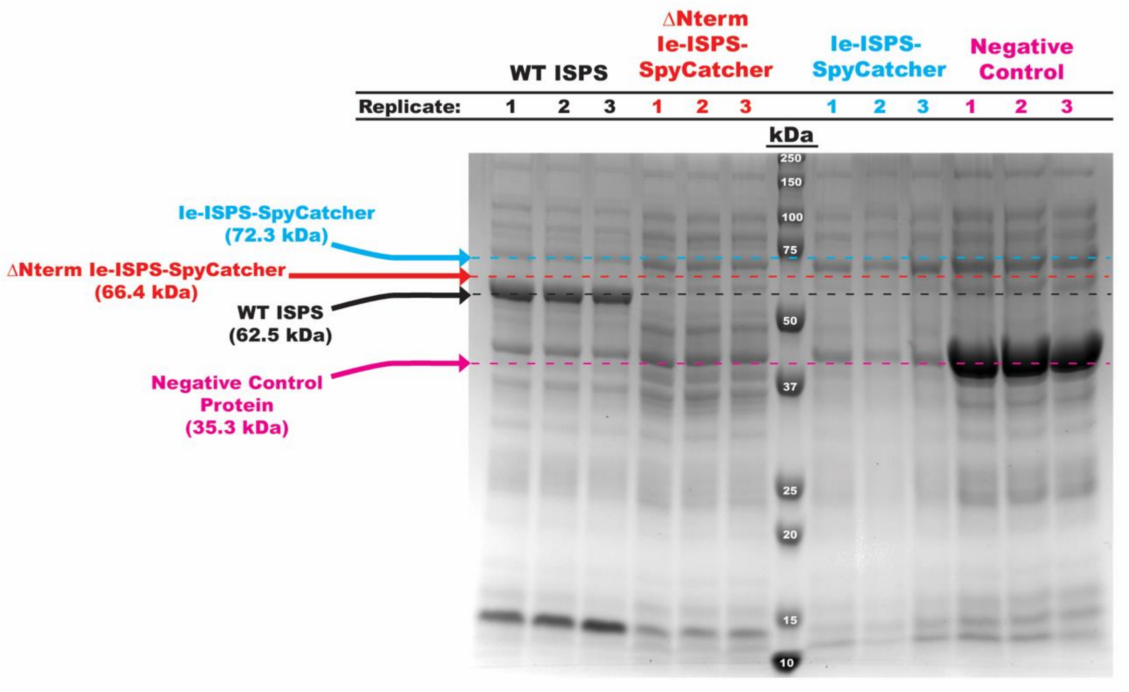

Supplementary Figure S1. ISPS abundance in clarified *E. coli* cell lysates. SDS-PAGE gel of whole cell lysates from *E. coli* strains expressing different ISPS variants. Coomassie Blue stained gel of whole cell lysates from strains expressing WT ISPS (black), ΔNterm Ie-ISPS-SpyCatcher (red), Ie-ISPS-SpyCatcher (cyan), or a negative control (magenta). 4 µg of total protein was loaded per well for three replicates of each strain. Predicted molecular weights for each ISPS variant and the negative control protein are: WT ISPS (62.5 kDa), ΔNterm Ie-ISPS-SpyCatcher (66.4 kDa), Ie-ISPS-SpyCatcher (72.3 kDa), and the negative control protein (35.3 kDa). Molecular weights were calculated using Benchling (https://benchling.com). Bio-Rad Precision Plus Dual Color Standards were used to estimate molecular weights in the gel.

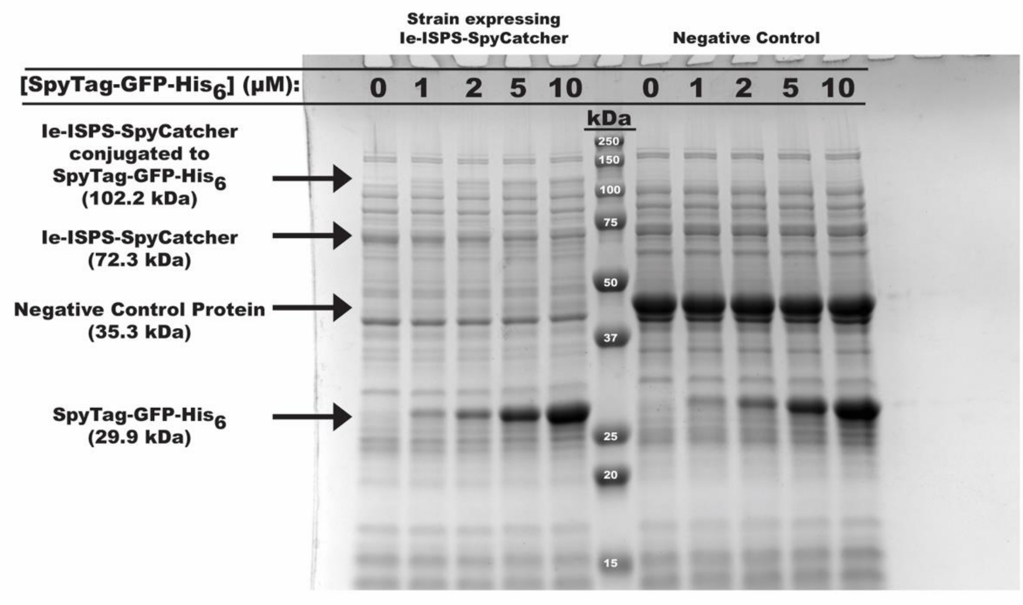

Supplementary Figure S2. Uncropped gel image of SpyTag-GFP-His$_6$ conjugation reactions. Uncropped image of the Coomassie Blue stained gel used in Figure 4. Predicted molecular weights for individual and conjugated proteins: Ie-ISPS-SpyCatcher (72.3 kDa), SpyTag-GFP-His$_6$ (29.9 kDa), Ie-ISPS-SpyCatcher conjugated with SpyTag-GFP-His$_6$ (102.2 kDa), and a negative control protein (35.3 kDa). Molecular weights were calculated using Benchling (https://benchling.com). Bio-Rad Precision Plus Dual Color Standards were used to estimate molecular weights in the gel.

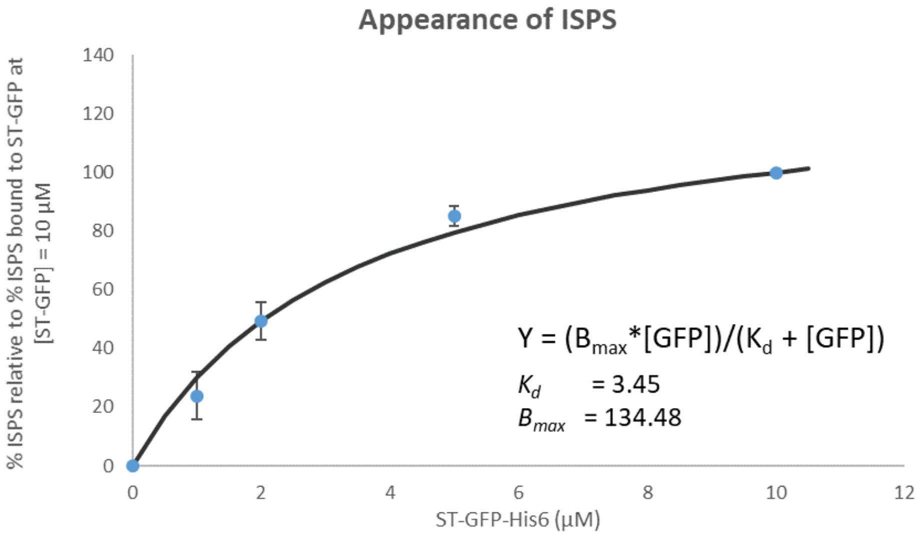

Supplementary Figure S3. The plot of appearance of SpyTag-GFP-His6 + Ie-ISPS-SpyCatcher fusion protein. The % of SpyTag-GFP-His6 + Ie-ISPS-SpyCatcher fusion protein (% ISPS) expressed as a % of the SpyTag-GFP-His6 + Ie-ISPS-SpyCatcher fusion protein detected with 10 µM of SpyTag-GFP-His6, is plotted against the concentration of SpyTag-GFP-His6 added to each corresponding conjugation mixture. The values presented in the graph represent mean±SE of three estimates of % SpyTag-GFP-His6 + Ie-ISPS-SpyCatcher fusion protein obtained from three independent conjugation reactions carried out using three independent lysates. The data was fitted with a rectangular hyperbola (Y = B_{max}*[GFP]/(K_d + [GFP]) to estimate B_{max} (maximum number of binding sites of Ie-ISPS-SpyCatcher) and K_d (dissociation constant at equilibrium).

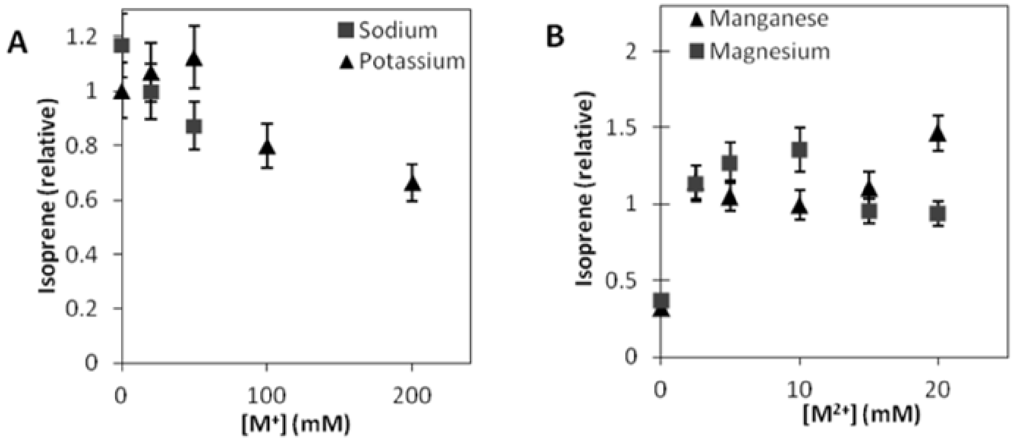

Supplementary Figure S4. Response of Ie-ISPS-GFP to sodium and potassium ion concentrations (A) and manganese and magnesium (B). In A, data is presented relative to potassium at 0 mM and in B, relative to 1 mM. Averages with standard error shown (n=3).

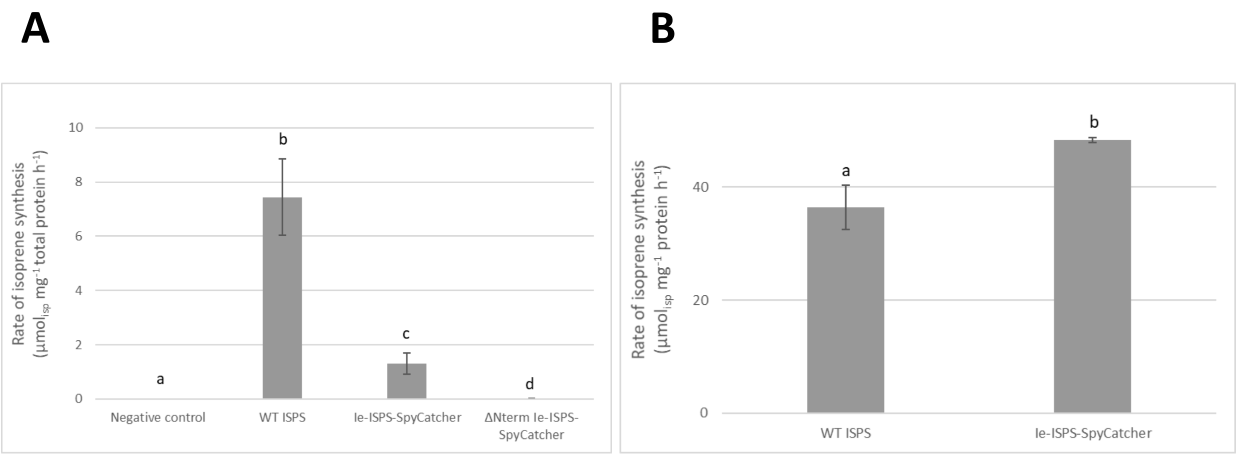

Supplementary Figure S5. Isoprene synthase activity of Ie-ISPS-SpyCatcher. (A) Isoprene synthase activity, measured as µmol of isoprene produced per mg of total protein per h, in lysates from cells expressing Ie-ISPS-SpyCatcher is compared with a negative control protein expressed from the same vector as all ISPS variants, WT ISPS, and a truncated form of Ie-ISPS-SpyCatcher lacking the N-terminal alpha helix preceding the γ-site (ΔNterm Ie-ISPS-SpyCatcher). (B) Isoprene synthase activity measured as µmol of isoprene produced per mg of either the WT ISPS or Ie-ISPS-SpyCatcher protein per h, respectively. 1 mM DMADP was used in the assay. Averages with standard errors are shown (n=3). Different lower case letters signify statistically significant differences between means at α = 0.05.

Supplementary Table S1. Amino acid sequences for proteins used in this study

Protein	Amino Acid Sequence	Comments
WT ISPS	MARRSANYEPNSWDYDYLLSSDTDESIEVYKDKA<br>KKLEAEVRREINNEKAEFLTLLELIDNVQRLGLG<br>YRFESDIRGALDRFVSSGGFDAVTKTSLHGTALS<br>FRLLRQHGFEVSQEAFSGFKDQNGNFLENLKEDI<br>KAILSLYEASFLALEGENILDEAKVFAISHLKEL<br>SEEKIGKELAEQVNHALELPLHRRTQRLEAVWSI<br>EAYRKKEDANQVLLELAILDYNMIQSVYQRDLRE<br>TSRWWRRVGLATKLHFARDRLIESFYWAVGVAFE<br>PQYSDCRNSVAKMFSFVTIIDDIYDVYGTLDELE<br>LFTDAVERWDVNAINDLPDYMKLCFLALYNTINE<br>IAYDNLKDKGENILPYLTKAWADLCNAFLQEAKW<br>LYNKSTPTFDDYFGNAWKSSSGPLQLVFAYFAVV<br>QNIKKEEIENLQKYHDTISRPSHIFRLCNDLASA<br>SAEIARGETANSVSCYMRTKGISEELATESVMNL<br>IDETWKKMNKEKLGGSLFAKPFVETAINLARQSH<br>CTYHNGDAHTSPDELTRKRVLSVITEPILPFER	
Ie-ISPS-GFP	MARRSANYEPNSWDYDYLLSSDTDESIEVYKDKA<br>KKLEAEVRREINNGSGSGSGSGS**SKGEELFTGVV**<br>**PILVELDGDVNGHKFSVSGEGEGDATYGKLTLKF**<br>**ICTTGKLPVPWPTLVTTLTYGVQCFARYPDHMKQ**<br>**HDFFKSAMPEGYVQERTIFFKDDGNYKTRAEVKF**<br>**EGDTLVNRIELKGIDFKEDGNILGHKLEYNYNSH**<br>**KVYITADKQKNGIKVNFKTRHNIEDGSVQLADHY**<br>**QQNTPIGDGPVLLPDNHYLSTQSALSKDPNEKRD**<br>**HMVLLEFVTAAGITLGMDELYK**GSGSGSGSRSEK<br>AEFLTLLELIDNVQRLGLGYRFESDIRGALDRFV<br>SSGGFDAVTKTSLHGTALSFRLLRQHGFEVSQEA<br>FSGFKDQNGNFLENLKEDIKAILSLYEASFLALE<br>GENILDEAKVFAISHLKELSEEKIGKELAEQVNH<br>ALELPLHRRTQRLEAVWSIEAYRKKEDANQVLLE<br>LAILDYNMIQSVYQRDLRETSRWWRRVGLATKLH<br>FARDRLIESFYWAVGVAFEPQYSDCRNSVAKMFS<br>FVTIIDDIYDVYGTLDELELFTDAVERWDVNAIN<br>DLPDYMKLCFLALYNTINEIAYDNLKDKGENILP<br>YLTKAWADLCNAFLQEAKWLYNKSTPTFDDYFGN<br>AWKSSSGPLQLVFAYFAVVQNIKKEEIENLQKYH<br>DTISRPSHIFRLCNDLASASAEIARGETANSVSC<br>YMRTKGISEELATESVMNLIDETWKKMNKEKLGG<br>SLFAKPFVETAINLARQSHCTYHNGDAHTSPDEL<br>TRKRVLSVITEPILPFER	GFP sequence in bold.

SUMO-Ie-ISPS-GFP	MGSSWSHPQFEKGSGLVPRGSASMSDSEVNQEAK<br>PEVKPEVKPETHINLKVSDGSSEIFFKIKKTTPL<br>RRLMEAFAKRQGKEMDSLRFLYDGIRIQADQTPE<br>DLDMEDNDIIEAHREQIGGMTEARRSANYEPNSW<br>DYDYLLSSDTDESIEVYKDKAKKLEAEVRREINN<br>GSGSGSGSGS**SKGEELFTGVVPILVELDGDVNGH**<br>**KFSVSGEGEGDATYGKLTLKFICTTGKLPVPWPT**<br>**LVTTLTYGVQCFARYPDHMKQHDFFKSAMPEGYV**<br>**QERTIFFKDDGNYKTRAEVKFEGDTLVNRIELKG**<br>**IDFKEDGNILGHKLEYNYNSHKVYITADKQKNGI**<br>**KVNFKTRHNIEDGSVQLADHYQQNTPIGDGPVLL**<br>**PDNHYLSTQSALSKDPNEKRDHMVLLEFVTAAGI**<br>**TLGMDELYK**GSGSGSGSRSEKAEFLTLLELIDNV<br>QRLGLGYRFESDIRGALDRFVSSGGFDAVTKTSL<br>HGTALSFRLLRQHGFEVSQEAFSGFKDQNGNFLE<br>NLKEDIKAILSLYEASFLALEGENILDEAKVFAI<br>SHLKELSEEKIGKELAEQVNHALELPLHRRTQRL<br>EAVWSIEAYRKKEDANQVLLELAILDYNMIQSVY<br>QRDLRETSRWWRRVGLATKLHFARDRLIESFYWA<br>VGVAFEPQYSDCRNSVAKMFSFVTIIDDIYDVYG<br>TLDELELFTDAVERWDVNAINDLPDYMKLCFLAL<br>YNTINEIAYDNLKDKGENILPYLTKAWADLCNAF<br>LQEAKWLYNKSTPTFDDYFGNAWKSSSGPLQLVF<br>AYFAVVQNIKKEEIENLQKYHDTISRPSHIFRLC<br>NDLASASAEIARGETANSVSCYMRTKGISEELAT<br>ESVMNLIDETWKKMNKEKLGGSLFAKPFVETAIN<br>LARQSHCTYHNGDAHTSPDELTRKRVLSVITEPI<br>LPFER	SUMO-Smt3 underlined and GFP sequence in bold.
SpyTag-GFP-His$_6$	MGEL**AHIVMVDAYKPTK**GSMRKGEELFTGVVPIL<br>VELDGDVNGHKFSVRGEGEGDATNGKLTLKFICT<br>TGKLPVPWPTLVTTLTYGVQCFARYPDHMKQHDF<br>FKSAMPEGYVQERTISFKDDGTYKTRAEVKFEGD<br>TLVNRIELKGIDFKEDGNILGHKLEYNFNSHNVY<br>ITADKQKNGIKANFKIRHNVEDGSVQLADHYQQN<br>TPIGDGPVLLPDNHYLSTQSVLSKDPNEKRDHMV<br>LLEFVTAAGITHGMDELYKLE**HHHHHH**	N-terminal SpyTag and C-terminal His$_6$ sequences are in bold.
ΔNterm Ie-ISPS-SpyCatcher	M**DSATHIKFSKRDEDGKELAGATMELRDSSGKTI**<br>**STWISDGQVKDFYLYPGKYTFVETAAPDGYEVAT**<br>**AITFTVNEQGQVTVN**GGSGGSEKAEFLTLLELID<br>NVQRLGLGYRFESDIRGALDRFVSSGGFDAVTKT<br>SLHGTALSFRLLRQHGFEVSQEAFSGFKDQNGNF<br>LENLKEDIKAILSLYEASFLALEGENILDEAKVF<br>AISHLKELSEEKIGKELAEQVNHALELPLHRRTQ<br>RLEAVWSIEAYRKKEDANQVLLELAILDYNMIQS<br>VYQRDLRETSRWWRRVGLATKLHFARDRLIESFY	SpyCatcher is in bold, GlySer linker is underlined.

	WAVGVAFEPQYSDCRNSVAKMFSFVTIIDDIYDV<br>YGTLDELELFTDAVERWDVNAINDLPDYMKLCFL<br>ALYNTINEIAYDNLKDKGENILPYLTKAWADLCN<br>AFLQEAKWLYNKSTPTFDDYFGNAWKSSSGPLQL<br>VFAYFAVVQNIKKEEIENLQKYHDTISRPSHIFR<br>LCNDLASASAEIARGETANSVSCYMRTKGISEEL<br>ATESVMNLIDETWKKMNKEKLGGSLFAKPFVETA<br>INLARQSHCTYHNGDAHTSPDELTRKRVLSVITE<br>PILPFER	
Ie-ISPS-SpyCatcher	MARRSANYEPNSWDYDYLLSSDTDESIEVYKDKA<br>KKLEAEVRREINNGGSGGS**DSATHIKFSKRDEDG**<br>**KELAGATMELRDSSGKTISTWISDGQVKDFYLYP**<br>**GKYTFVETAAPDGYEVATAITFTVNEQGQVTVN**G<br>GSGGSEKAEFLTLLELIDNVQRLGLGYRFESDIR<br>GALDRFVSSGGFDAVTKTSLHGTALSFRLLRQHG<br>FEVSQEAFSGFKDQNGNFLENLKEDIKAILSLYE<br>ASFLALEGENILDEAKVFAISHLKELSEEKIGKE<br>LAEQVNHALELPLHRRTQRLEAVWSIEAYRKKED<br>ANQVLLELAILDYNMIQSVYQRDLRETSRWWRRV<br>GLATKLHFARDRLIESFYWAVGVAFEPQYSDCRN<br>SVAKMFSFVTIIDDIYDVYGTLDELELFTDAVER<br>WDVNAINDLPDYMKLCFLALYNTINEIAYDNLKD<br>KGENILPYLTKAWADLCNAFLQEAKWLYNKSTPT<br>FDDYFGNAWKSSSGPLQLVFAYFAVVQNIKKEEI<br>ENLQKYHDTISRPSHIFRLCNDLASASAEIARGE<br>TANSVSCYMRTKGISEELATESVMNLIDETWKKM<br>NKEKLGGSLFAKPFVETAINLARQSHCTYHNGDA<br>HTSPDELTRKRVLSVITEPILPFER	SpyCatcher is in bold, insulating GlySer linkers underlined.

Supplementary Sequence S1. DNA Sequence of the pD881/Ie-ISPS-GFP plasmid.

CCCCTTGGGGCCTCTAAACGGGTCTTGAGGGGTTTTTTGCTGAAAGGAGGAACTATATCCGGGTAACGAA
TTCAAGCTTGATATCATTCAGGACGAGCCTCAGACTCCAGCGTAACTGGACTGCAATCAACTCACTGGCT
CACCTTCACGGGTGGGCCTTTCTTCGGTAGAAGTCTTCTTAATAAGATGATCTTCTTGAGATCGTTTTGG
TCTGCGCGTAATCTCTTGCTCTGAAAACGAAAAAACCGCCTTGCAGGGCGGTTTTTCGAAGGTTCTCTGA
GCTACCAACTCTTTGAACCGAGGTAACTGGCTTGGAGGAGCGCAGTCACCAAAACTTGTCCTTTCAGTTT
AGCCTTAACCGGCGCATGACTTCAAGACTAACTCCTCTAAATCAATTACCAGTGGCTGCTGCCAGTGGTG
CTTTTGCATGTCTTTCCGGGTTGGACTCAAGACGATAGTTACCGGATAAGGCGCAGCGGTCGGACTGAAC
GGGGGGTTCGTGCATACAGTCCAGCTTGGAGCGAACTGCCTACCCGGAACTGAGTGTCAGGCGTGGAATG
AGACAAACGCGGCCATAACAGCGGAATGACACCGGTAAACCGAAAGGCAGGAACAGGAGAGCGCACGAGG
GAGCCGCCAGGGGGAAACGCCTGGTATCTTTATAGTCCTGTCGGGTTTCGCCACCACTGATTTGAGCGTC
AGATTTCGTGATGCTTGTCAGGGGGGCGGAGCCTATGGAAAAACGGCTTTGCCGCGGCCCTCTCACTTCC
CTGTTAAGTATCTTCCTGGCATCTTCCAGGAAATCTCCGCCCCGTTCGTAAGCCATTTCCGCTCGCCGCA
GTCGAACGACCGAGCGTAGCGAGTCAGTGAGCGAGGAAGCGGAATATATCCTGTATCACATATTCTGCTG
ACGCACCGGTGCAGCCTTTTTTCTCCTGCCACATGAAGCACTTCACTGACACCCTCATCAGTGCCAACAT
AGTAAGCCAGTATACACTCCGCTAGCGCAGAAAGGCCCACCCGAAGGTGAGCCAGGTGATTACATTTGGG
CCCTCATTAGAAAAACTCATCGAGCATCAAATGAAATTGCAATTTATTCATATCAGGATTATCAATACCA
TATTTTTGAAAAAGCCGTTTCTGTAATGAAGGAGAAAACTCACCGAGGCAGTTCCATAGGATGGCAAGAT
CCTGGTATCGGTCTGCGATTCCGACTCGTCCAACATCAATACAACCTATTAATTTCCCCTCGTCAAAAAT
AAGGTTATCAAGTGAGAAATCACCATGAGTGACGACTGAATCCGGTGAGAATGGCAAAAGTTTATGCATT
TCTTTCCAGACTTGTTCAACAGGCCAGCCATTACGCTCGTCATCAAAATCACTCGCATCAACCAAACCGT
TATTCATTCGTGATTGCGCCTGAGCGAGGCGAAATACGCGATCGCTGTTAAAAGGACAATTACAAACAGG
AATCGAGTGCAACCGGCGCAGGAACACTGCCAGCGCATCAACAATATTTTCACCTGAATCAGGATATTCT
TCTAATACCTGGAACGCTGTTTTTCCGGGGATCGCAGTGGTGAGTAACCATGCATCATCAGGAGTACGGA
TAAAATGCTTGATGGTCGGAAGTGGCATAAATTCCGTCAGCCAGTTTAGTCTGACCATCTCATCTGTAAC
ATCATTGGCAACGCTACCTTTGCCATGTTTCAGAAACAACTCTGGCGCATCGGGCTTCCCATACAAGCGA
TAGATTGTCGCACCTGATTGCCCGACATTATCGCGAGCCCATTTATACCCATATAAATCAGCATCCATGT
TGGAATTTAATCGCGGCCTCGACGTTTCCCGTTGAATATGGCTCATAGCTCCTGAAAATCTCGATAACTC
AAAAAATACGCCCGGTAGTGATCTTATTTCATTATGGTGAAAGTTGGAACCTCTTACGTGCCGATCAAGA
AGACGGTCAAAAGCCTCCGGTCGGAGGCTTTTGACTTTCTGCTATGGAGGTCAGGTATGATTTAAATGGT
CAGTATTGAGCGATATCTAGAGAATTCGTCCACCACAATTCAGCAAATTGTGAACATCATCACGTTCATC
TTTCCCTGGTTGCCAATGGCCCATTTTCCTGTCAGTAACGAGAAGGTCGCGAATTCAGGCGCTTTTTAGA
CTGGTCGTAGAGACCATGAAATTCTTTTAAGGAGGTAAAAAATGGCCCGTCGCAGTGCTAACTATGAGCC
GAATTCCTGGGACTATGACTATCTGTTGAGTTCCGACACCGATGAAAGCATCGAGGTGTATAAAGACAAA
GCGAAAAAGCTTGAAGCGGAAGTGCGTCGTGAGATCAACAACGGCAGTGGATCAGGTTCGGGCTCTGGAT
CCAGTAAAGGCGAAGAACTGTTTACGGGAGTGGTTCCTATCTTGGTGGAGCTTGATGGAGACGTGAACGG
ACACAAATTTTCCGTAAGCGGGGAAGGCGAAGGTGATGCTACCTATGGCAAACTTACTCTGAAATTTATA
TGTACTACCGGAAAATTGCCAGTGCCCTGGCCAACGTTGGTCACAACTTTGACTTATGGAGTACAGTGCT
TTGCGCGGTACCCAGACCACATGAAACAGCATGATTTTTTCAAGAGTGCAATGCCGGAAGGTTATGTTCA
GGAGAGAACCATATTTTTCAAGGACGACGGAAACTATAAGACCAGAGCCGAAGTGAAATTCGAGGGAGAT
ACTTTAGTTAATAGAATAGAATTGAAAGGCATCGATTTCAAGGAGGATGGAAACATCCTTGGACATAAGC
TTGAATACAACTATAACAGCCACAAGGTTTACATCACTGCCGACAAGCAGAAAAACGGCATAAAAGTCAA
CTTCAAAACCCGGCATAATATTGAAGATGGGTCAGTGCAATTAGCAGATCATTACCAACAGAATACACCT
ATAGGGGACGGCCCGGTTTTGTTGCCTGATAATCATTACCTTAGTACACAGTCGGCCTTGTCCAAAGATC
CCAACGAAAAGCGCGATCACATGGTACTGCTGGAATTCGTGACTGCAGCCGGAATTACGTTGGGGATGGA

TGAACTTTATAAGGGCTCCGGCAGTGGGAGTGGGTCCAGATCTGAAAAGGCAGAGTTTTTGACATTATTG
GAGCTTATAGATAATGTCCAGCGCTTGGGATTGGGTTACCGGTTCGAAAGCGACATCCGGGGGGCCTTGG
ATAGATTTGTGTCCAGCGGAGGTTTCGATGCGGTGACGAAGACTAGCTTGCACGGCACCGCATTGTCTTT
CCGTTTATTGCGTCAGCACGGGTTCGAAGTCTCACAGGAAGCCTTTTCTGGTTTTAAGGATCAGAATGGG
AATTTCCTTGAGAACCTTAAAGAGGACATCAAGGCAATTTTAAGTTTGTATGAAGCTTCGTTTTTAGCAC
TGGAGGGCGAAAACATATTGGACGAAGCTAAGGTGTTCGCTATCTCGCATCTGAAGGAGCTTTCAGAAGA
AAAGATCGGCAAGGAATTAGCCGAGCAGGTGAATCACGCGCTTGAGCTGCCCCTTCATCGCAGAACGCAA
CGTCTTGAAGCGGTATGGAGTATCGAGGCGTATCGTAAAAAAGAGGACGCTAACCAAGTTTTATTAGAAT
TAGCGATCTTGGACTATAATATGATTCAGAGTGTGTATCAAAGAGACCTTCGGGAGACTTCACGCTGGTG
GCGGAGAGTAGGCTTGGCTACTAAGCTTCATTTCGCACGCGATCGGTTAATAGAGTCGTTTTACTGGGCG
GTTGGTGTTGCATTCGAACCCCAATATTCGGATTGTAGAAATTCCGTAGCCAAAATGTTCAGCTTCGTAA
CGATTATAGATGACATCTATGATGTCTACGGTACTCTGGACGAGCTGGAATTGTTTACGGATGCAGTTGA
GCGCTGGGATGTCAACGCTATTAACGATCTGCCTGACTACATGAAGTTATGCTTTCTTGCCCTTTACAAC
ACCATCAATGAGATAGCTTACGACAACCTGAAAGATAAGGGAGAGAACATATTACCATATTTGACAAAGG
CCTGGGCAGACTTATGCAATGCGTTTCTTCAGGAGGCAAAGTGGCTTTACAACAAAAGTACGCCAACGTT
CGACGACTACTTCGGTAATGCGTGGAAGTCCAGTTCTGGACCCCTTCAGTTAGTGTTCGCGTACTTCGCG
GTAGTCCAAAACATCAAGAAGGAGGAGATCGAAAATTTACAAAAGTACCATGACACGATTTCACGTCCCA
GCCACATTTTTCGTTTATGCAATGATCTGGCCTCCGCCTCTGCAGAAATAGCACGGGGAGAGACCGCTAA
TTCAGTATCCTGCTACATGCGGACAAAGGGGATATCGGAAGAACTTGCGACAGAAAGCGTTATGAACCTG
ATCGACGAGACCTGGAAGAAAATGAACAAAGAAAAGTTAGGAGGTTCCCTTTTTGCAAAGCCATTTGTTG
AAACTGCGATTAATTTGGCAAGACAGTCACACTGTACCTACCACAACGGAGATGCTCACACGAGCCCAGA
TGAGCTTACACGCAAGAGAGTTCTTTCGGTCATCACAGAACCTATTCTGCCATTTGAGCGTtaaGCGGCC
GCCACCGCTGAGCAATAACTAGCATAA

Supplementary Sequence S2. DNA Sequence of the pD881/SUMO-Ie-ISPS-GFP plasmid.

ATCAAATGAAATTGCAATTTATTCATATCAGGATTATCAATACCATATTTTTGAAAAAGCCGTTTCTGTA
ATGAAGGAGAAAACTCACCGAGGCAGTTCCATAGGATGGCAAGATCCTGGTATCGGTCTGCGATTCCGAC
TCGTCCAACATCAATACAACCTATTAATTTCCCCTCGTCAAAAATAAGGTTATCAAGTGAGAAATCACCA
TGAGTGACGACTGAATCCGGTGAGAATGGCAAAAGTTTATGCATTTCTTTCCAGACTTGTTCAACAGGCC
AGCCATTACGCTCGTCATCAAAATCACTCGCATCAACCAAACCGTTATTCATTCGTGATTGCGCCTGAGC
GAGGCGAAATACGCGATCGCTGTTAAAAGGACAATTACAAACAGGAATCGAGTGCAACCGGCGCAGGAAC
ACTGCCAGCGCATCAACAATATTTTCACCTGAATCAGGATATTCTTCTAATACCTGGAACGCTGTTTTTC
CGGGGATCGCAGTGGTGAGTAACCATGCATCATCAGGAGTACGGATAAAATGCTTGATGGTCGGAAGTGG
CATAAATTCCGTCAGCCAGTTTAGTCTGACCATCTCATCTGTAACATCATTGGCAACGCTACCTTTGCCA
TGTTTCAGAAACAACTCTGGCGCATCGGGCTTCCCATACAAGCGATAGATTGTCGCACCTGATTGCCCGA
CATTATCGCGAGCCCATTTATACCCATATAAATCAGCATCCATGTTGGAATTTAATCGCGGCCTCGACGT
TTCCCGTTGAATATGGCTCATAGCTCCTGAAAATCTCGATAACTCAAAAAATACGCCCGGTAGTGATCTT
ATTTCATTATGGTGAAAGTTGGAACCTCTTACGTGCCGATCAAGAAGACGGTCAAAAGCCTCCGGTCGGA
GGCTTTTGACTTTCTGCTATGGAGGTCAGGTATGATTTAAATGGTCAGTATTGAGCGATATCTAGAGAAT
TCGTCCACCACAATTCAGCAAATTGTGAACATCATCACGTTCATCTTTCCCTGGTTGCCAATGGCCCATT
TTCCTGTCAGTAACGAGAAGGTCGCGAATTCAGGCGCTTTTTAGACTGGTCGTAGAGACCATGAAATTCT
TTTAAGGAGGTAAAAAATGGGCAGCAGCTGGAGCCATCCGCAGTTTGAAAAAGGCAGCGGCCTGGTGCCG
CGCGGCAGCGCAAGCATGTCGGACTCAGAAGTCAATCAAGAAGCTAAGCCAGAGGTCAAGCCAGAAGTCA
AGCCTGAGACTCACATCAATTTAAAGGTGTCCGATGGATCTTCAGAGATCTTCTTCAAGATCAAAAAGAC
CACTCCTTTAAGAAGGCTGATGGAAGCGTTCGCTAAAAGACAGGGTAAGGAAATGGACTCCTTAAGATTC
TTGTACGACGGTATTCGTATTCAAGCTGATCAGACCCCTGAAGATTTGGACATGGAGGATAACGATATTA
TTGAGGCTCACAGAGAACAGATTGGTGGTATGACGGAAGCGCGCCGTTCCGCTAACTATGAGCCAAATTC
ATGGGATTACGATTATCTTCTGAGCTCAGATACGGACGAATCAATCGAAGTGTATAAAGATAAGGCCAAG
AAACTGGAAGCTGAGGTCCGTCGCGAAATTAATAACGGCAGTGGATCAGGTTCGGGCTCTGGATCCAGTA
AAGGCGAAGAACTGTTTACGGGAGTGGTTCCTATCTTGGTGGAGCTTGATGGAGACGTGAACGGACACAA
ATTTTCCGTAAGCGGGGAAGGCGAAGGTGATGCTACCTATGGCAAACTTACTCTGAAATTTATATGTACT
ACCGGAAAATTGCCAGTGCCCTGGCCAACGTTGGTCACAACTTTGACTTATGGAGTACAGTGCTTTGCGC
GGTACCCAGACCACATGAAACAGCATGATTTTTTCAAGAGTGCAATGCCGGAAGGTTATGTTCAGGAGAG
AACCATATTTTTCAAGGACGACGGAAACTATAAGACCAGAGCCGAAGTGAAATTCGAGGGAGATACTTTA
GTTAATAGAATAGAATTGAAAGGCATCGATTTCAAGGAGGATGGAAACATCCTTGGACATAAGCTTGAAT
ACAACTATAACAGCCACAAGGTTTACATCACTGCCGACAAGCAGAAAAACGGCATAAAAGTCAACTTCAA
AACCCGGCATAATATTGAAGATGGGTCAGTGCAATTAGCAGATCATTACCAACAGAATACACCTATAGGG
GACGGCCCGGTTTTGTTGCCTGATAATCATTACCTTAGTACACAGTCGGCCTTGTCCAAAGATCCCAACG
AAAAGCGCGATCACATGGTACTGCTGGAATTCGTGACTGCAGCCGGAATTACGTTGGGGATGGATGAACT
TTATAAGGGCTCCGGCAGTGGGAGTGGGTCCAGATCTGAGAAAGCAGAGTTTCTGACGCTGCTTGAACTC
ATCGACAATGTGCAGCGTCTGGGCCTGGGGTATCGTTTTGAGTCGGACATTCGCGGTGCCTTGGATCGTT
TTGTCAGTTCTGGAGGATTTGATGCAGTAACGAAAACGAGTCTCCACGGCACGGCGCTGAGCTTTCGCTT
GCTGCGCCAACACGGCTTTGAGGTTTCTCAGGAAGCGTTTTCTGGCTTTAAGGACCAAAATGGTAATTTT
CTGGAGAACTTGAAAGAAGATATTAAAGCGATCCTGAGCTTATACGAGGCGAGTTTCCTGGCCCTCGAGG
GCGAAAACATTCTGGACGAAGCTAAAGTTTTTGCGATTTCCCACCTCAAAGAGTTGTCAGAAGAAAAAAT
TGGCAAAGAGCTGGCAGAACAGGTCAATCACGCGTTAGAATTGCCGCTGCACCGCCGTACCCAACGCCTG
GAAGCTGTATGGTCAATCGAAGCATATCGTAAAAAGGAGGACGCGAATCAGGTGTTGCTGGAACTGGCCA
TCCTGGACTATAACATGATTCAGTCGGTGTACCAGCGCGATCTGCGCGAAACGTCTCGCTGGTGGCGTCG
TGTGGGGCTGGCGACTAAGCTCCATTTTGCTCGCGACCGTCTGATCGAAAGCTTTTATTGGGCGGTTGGA

GTGGCTTTTGAACCTCAGTATAGCGATTGTCGCAACTCCGTTGCTAAAATGTTCAGTTTTGTGACCATCA
TCGACGACATTTACGATGTATACGGTACCCTCGACGAACTGGAACTGTTTACCGACGCTGTGGAACGGTG
GGATGTGAATGCGATCAACGACCTCCCGGATTATATGAAATTATGTTTCTTAGCCCTGTACAATACCATC
AACGAAATTGCATACGACAACCTGAAAGATAAAGGGGAGAATATCTTGCCTTACCTCACCAAAGCTTGGG
CCGATCTTTGCAACGCGTTCTTACAAGAAGCCAAATGGTTATATAATAAGTCCACCCCGACCTTCGACGA
CTACTTCGGGAACGCTTGGAAGAGCTCGAGCGGCCCTCTTCAGCTGGTGTTTGCGTATTTTGCAGTGGTG
CAGAACATCAAAAAGGAAGAAATCGAAAACCTTCAGAAATACCACGATACGATTTCGCGGCCATCGCATA
TTTTCCGCCTGTGTAATGATTTGGCGAGCGCCTCCGCGGAAATCGCACGCGGCGAAACCGCTAACAGCGT
GAGCTGTTACATGCGCACAAAGGGCATTAGCGAGGAACTGGCCACAGAGTCTGTCATGAATCTGATCGAT
GAGACGTGGAAAAAAATGAACAAGGAAAAACTGGGTGGCAGCCTCTTTGCTAAACCATTCGTCGAAACGG
CGATTAACCTGGCCCGCCAGAGCCACTGCACCTACCACAATGGCGATGCCCACACGAGCCCGGACGAACT
GACTCGTAAGCGTGTTCTGAGCGTGATCACGGAACCGATCCTGCCTTTTGAACGTTAAGGTTAGGGTCTC
GCGGCCGCCACCGCTGAGCAATAACTAGCATAACCCCTTGGGGCCTCTAAACGGGTCTTGAGGGGTTTTT
TGCTGAAAGGAGGAACTATATCCGGGTAACGAATTCAAGCTTGATATCATTCAGGACGAGCCTCAGACTC
CAGCGTAACTGGACTGCAATCAACTCACTGGCTCACCTTCACGGGTGGGCCTTTCTTCGGTAGAAGTCTT
CTTAATAAGATGATCTTCTTGAGATCGTTTTGGTCTGCGCGTAATCTCTTGCTCTGAAAACGAAAAAACC
GCCTTGCAGGGCGGTTTTTCGAAGGTTCTCTGAGCTACCAACTCTTTGAACCGAGGTAACTGGCTTGGAG
GAGCGCAGTCACCAAAACTTGTCCTTTCAGTTTAGCCTTAACCGGCGCATGACTTCAAGACTAACTCCTC
TAAATCAATTACCAGTGGCTGCTGCCAGTGGTGCTTTTGCATGTCTTTCCGGGTTGGACTCAAGACGATA
GTTACCGGATAAGGCGCAGCGGTCGGACTGAACGGGGGGTTCGTGCATACAGTCCAGCTTGGAGCGAACT
GCCTACCCGGAACTGAGTGTCAGGCGTGGAATGAGACAAACGCGGCCATAACAGCGGAATGACACCGGTA
AACCGAAAGGCAGGAACAGGAGAGCGCACGAGGGAGCCGCCAGGGGGAAACGCCTGGTATCTTTATAGTC
CTGTCGGGTTTCGCCACCACTGATTTGAGCGTCAGATTTCGTGATGCTTGTCAGGGGGGCGGAGCCTATG
GAAAAACGGCTTTGCCGCGGCCCTCTCACTTCCCTGTTAAGTATCTTCCTGGCATCTTCCAGGAAATCTC
CGCCCCGTTCGTAAGCCATTTCCGCTCGCCGCAGTCGAACGACCGAGCGTAGCGAGTCAGTGAGCGAGGA
AGCGGAATATATCCTGTATCACATATTCTGCTGACGCACCGGTGCAGCCTTTTTTCTCCTGCCACATGAA
GCACTTCACTGACACCCTCATCAGTGCCAACATAGTAAGCCAGTATACACTCCGCTAGCGCAGAAAGGCC
CACCCGAAGGTGAGCCAGGTGATTACATTTGGGCCCTCATTAGAAAAACTCATCGAGC

Supplementary Sequence S3. DNA sequence for the pBbE2K-Ie-ISPS-SpyCatcher

CCGAATAAGAAGGCTGGCTCTGCACCTTGGTGATCAAATAATTCGATAGCTTGTCGTAATAATGGCGGCA
TACTATCAGTAGTAGGTGTTTCCCTTTCTTCTTTAGCGACTTGATGCTCTTGATCTTCCAATACGCAACC
TAAAGTAAAATGCCCCACAGCGCTGAGTGCATATAATGCATTCTCTAGTGAAAAACCTTGTTGGCATAAA
AAGGCTAATTGATTTTCGAGAGTTTCATACTGTTTTTCTGTAGGCCGTGTACCTAAATGTACTTTTGCTC
CATCGCGATGACTTAGTAAAGCACATCTAAAACTTTTAGCGTTATTACGTAAAAAATCTTGCCAGCTTTC
CCCTTCTAAAGGGCAAAAGTGAGTATGGTGCCTATCTAACATCTCAATGGCTAAGGCGTCGAGCAAAGCC
CGCTTATTTTTTACATGCCAATACAATGTAGGCTGCTCTACACCTAGCTTCTGGGCGAGTTTACGGGTTG
TTAAACCTTCGATTCCGACCTCATTAAGCAGCTCTAATGCGCTGTTAATCACTTTACTTTTATCTAATCT
AGACATCATTAATTCCTAATTTTTGTTGACACTCTATCGTTGATAGAGTTATTTTACCACTCCCTATCAG
TGATAGAGAAAAGAATTCAAAAGATCTTTTAAGAAGGAGATATACATATGGCACGTCGCAGTGCAAACTA
TGAGCCGAATTCCTGGGACTATGACTATCTGTTGAGTTCCGACACCGATGAAAGCATCGAGGTGTATAAA
GACAAAGCGAAAAAGCTTGAAGCGGAAGTGCGTCGTGAGATCAACAACGGTGGATCAGGTGGTAGTGATA
GTGCTACCCATATTAAATTCTCAAAACGTGATGAGGACGGCAAAGAGTTAGCTGGTGCAACTATGGAGTT
GCGTGATTCATCTGGTAAAACTATTAGTACATGGATTTCAGATGGACAAGTGAAAGATTTCTACCTGTAT
CCAGGAAAATATACATTTGTCGAAACCGCAGCACCAGACGGTTATGAGGTAGCAACTGCTATTACCTTTA
CAGTTAATGAGCAAGGTCAGGTTACTGTAAATGGAGGTAGCGGAGGAAGTGAAAAGGCAGAGTTTTTGAC
ATTATTGGAGCTTATAGATAATGTCCAGCGCTTGGGATTGGGTTACCGGTTCGAAAGCGACATCCGGGGG
GCCTTGGATAGATTTGTGTCCAGCGGAGGTTTCGATGCGGTGACGAAGACTAGCTTGCACGGCACCGCAT
TGTCTTTCCGTTTATTGCGTCAGCACGGGTTCGAAGTCTCACAGGAAGCCTTTTCTGGTTTTAAGGATCA
GAATGGGAATTTCCTTGAGAACCTTAAAGAGGACATCAAGGCAATTTTAAGTTTGTATGAAGCTTCGTTT
TTAGCACTGGAGGGCGAAAACATATTGGACGAAGCTAAGGTGTTCGCTATCTCGCATCTGAAGGAGCTTT
CAGAAGAAAAGATCGGCAAGGAATTAGCCGAGCAGGTGAATCACGCGCTTGAGCTGCCCCTTCATCGCAG
AACGCAACGTCTTGAAGCGGTATGGAGTATCGAGGCGTATCGTAAAAAAGAGGACGCTAACCAAGTTTTA
TTAGAATTAGCGATCTTGGACTATAATATGATTCAGAGTGTGTATCAAAGAGACCTTCGGGAGACTTCAC
GCTGGTGGCGGAGAGTAGGCTTGGCTACTAAGCTTCATTTCGCACGCGATCGGTTAATAGAGTCGTTTTA
CTGGGCGGTTGGTGTTGCATTCGAACCCCAATATTCGGATTGTAGAAATTCCGTAGCCAAAATGTTCAGC
TTCGTAACGATTATAGATGACATCTATGATGTCTACGGTACTCTGGACGAGCTGGAATTGTTTACGGATG
CAGTTGAGCGCTGGGATGTCAACGCTATTAACGATCTGCCTGACTACATGAAGTTATGCTTTCTTGCCCT
TTACAACACCATCAATGAGATAGCTTACGACAACCTGAAAGATAAGGGAGAGAACATATTACCATATTTG
ACAAAGGCCTGGGCAGACTTATGCAATGCGTTTCTTCAGGAGGCAAAGTGGCTTTACAACAAAAGTACGC
CAACGTTCGACGACTACTTCGGTAATGCGTGGAAGTCCAGTTCTGGACCCCTTCAGTTAGTGTTCGCGTA
CTTCGCGGTAGTCCAAAACATCAAGAAGGAGGAGATCGAAAATTTACAAAAGTACCATGACACGATTTCA
CGTCCCAGCCACATTTTTCGTTTATGCAATGATCTGGCCTCCGCCTCTGCAGAAATAGCACGGGGAGAGA
CCGCTAATTCAGTATCCTGCTACATGCGGACAAAGGGGATATCGGAAGAACTTGCGACAGAAAGCGTTAT
GAACCTGATCGACGAGACCTGGAAGAAAATGAACAAAGAAAAGTTAGGAGGTTCCCTTTTTGCAAAGCCA
TTTGTTGAAACTGCGATTAATTTGGCAAGACAGTCACACTGTACCTACCACAACGGAGATGCTCACACGA
GCCCAGATGAGCTTACACGCAAGAGAGTTCTTTCGGTCATCACAGAACCTATTCTGCCATTTGAGCGTTA
AGGATCCAAACTCGAGTAAGGATCTCCAGGCATCAAATAAAACGAAAGGCTCAGTCGAAAGACTGGGCCT
TTCGTTTTATCTGTTGTTTGTCGGTGAACGCTCTCTACTAGAGTCACACTGGCTCACCTTCGGGTGGGCC
TTTCTGCGTTTATACCTAGGGCGTTCGGCTGCGGCGAGCGGTATCAGCTCACTCAAAGGCGGTAATACGG
TTATCCACAGAATCAGGGGATAACGCAGGAAAGAACATGTGAGCAAAAGGCCAGCAAAAGGCCAGGAACC
GTAAAAAGGCCGCGTTGCTGGCGTTTTTCCATAGGCTCCGCCCCCCTGACGAGCATCACAAAAATCGACG
CTCAAGTCAGAGGTGGCGAAACCCGACAGGACTATAAAGATACCAGGCGTTTCCCCCTGGAAGCTCCCTC
GTGCGCTCTCCTGTTCCGACCCTGCCGCTTACCGGATACCTGTCCGCCTTTCTCCCTTCGGGAAGCGTGG

CGCTTTCTCATAGCTCACGCTGTAGGTATCTCAGTTCGGTGTAGGTCGTTCGCTCCAAGCTGGGCTGTGT
GCACGAACCCCCCGTTCAGCCCGACCGCTGCGCCTTATCCGGTAACTATCGTCTTGAGTCCAACCCGGTA
AGACACGACTTATCGCCACTGGCAGCAGCCACTGGTAACAGGATTAGCAGAGCGAGGTATGTAGGCGGTG
CTACAGAGTTCTTGAAGTGGTGGCCTAACTACGGCTACACTAGAAGGACAGTATTTGGTATCTGCGCTCT
GCTGAAGCCAGTTACCTTCGGAAAAAGAGTTGGTAGCTCTTGATCCGGCAAACAAACCACCGCTGGTAGC
GGTGGTTTTTTTGTTTGCAAGCAGCAGATTACGCGCAGAAAAAAAGGATCTCAAGAAGATCCTTTGATCT
TTTCTACGGGGTCTGACGCTCAGTGGAACGAAAACTCACGTTAAGGGATTTTGGTCATGACTAGTGCTTG
GATTCTCACCAATAAAAAACGCCCGGCGGCAACCGAGCGTTCTGAACAAATCCAGATGGAGTTCTGAGGT
CATTACTGGATCTATCAACAGGAGTCCAAGCGAGCTCTCGAACCCCAGAGTCCCGCTCAGAAGAACTCGT
CAAGAAGGCGATAGAAGGCGATGCGCTGCGAATCGGGAGCGGCGATACCGTAAAGCACGAGGAAGCGGTC
AGCCCATTCGCCGCCAAGCTCTTCAGCAATATCACGGGTAGCCAACGCTATGTCCTGATAGCGGTCCGCC
ACACCCAGCCGGCCACAGTCGATGAATCCAGAAAAGCGGCCATTTTCCACCATGATATTCGGCAAGCAGG
CATCGCCATGGGTCACGACGAGATCCTCGCCGTCGGGCATGCGCGCCTTGAGCCTGGCGAACAGTTCGGC
TGGCGCGAGCCCCTGATGCTCTTCGTCCAGATCATCCTGATCGACAAGACCGGCTTCCATCCGAGTACGT
GCTCGCTCGATGCGATGTTTCGCTTGGTGGTCGAATGGGCAGGTAGCCGGATCAAGCGTATGCAGCCGCC
GCATTGCATCAGCCATGATGGATACTTTCTCGGCAGGAGCAAGGTGAGATGACAGGAGATCCTGCCCCGG
CACTTCGCCCAATAGCAGCCAGTCCCTTCCCGCTTCAGTGACAACGTCGAGCACAGCTGCGCAAGGAACG
CCCGTCGTGGCCAGCCACGATAGCCGCGCTGCCTCGTCCTGCAGTTCATTCAGGGCACCGGACAGGTCGG
TCTTGACAAAAAGAACCGGGCGCCCCTGCGCTGACAGCCGGAACACGGCGGCATCAGAGCAGCCGATTGT
CTGTTGTGCCCAGTCATAGCCGAATAGCCTCTCCACCCAAGCGGCCGGAGAACCTGCGTGCAATCCATCT
TGTTCAATCATGCGAAACGATCCTCATCCTGTCTCTTGATCAGATCATGATCCCCTGCGCCATCAGATCC
TTGGCGGCAAGAAAGCCATCCAGTTTACTTTGCAGGGCTTCCCAACCTTACCAGAGGGCGCCCCAGCTGG
CAATTCCGACGTCTTAAGACCCACTTTCACATTTAAGTTGTTTTTCTAATCCGCATATAATCAATTCAAG
G

Supplementary Sequence S4. DNA sequence for the pBbE2K-SpyTag-GFP-His$_6$. Coding sequence for SpyTag-GFP-His$_6$ is indicated in bold.

GATCATCCTGATCGACAAGACCGGCTTCCATCCGAGTACGTGCTCGCTCGATGCGATGTTTCGCTTGGTG
GTCGAATGGGCAGGTAGCCGGATCAAGCGTATGCAGCCGCCGCATTGCATCAGCCATGATGGATACTTTC
TCGGCAGGAGCAAGGTGAGATGACAGGAGATCCTGCCCCGGCACTTCGCCCAATAGCAGCCAGTCCCTTC
CCGCTTCAGTGACAACGTCGAGCACAGCTGCGCAAGGAACGCCCGTCGTGGCCAGCCACGATAGCCGCGC
TGCCTCGTCCTGCAGTTCATTCAGGGCACCGGACAGGTCGGTCTTGACAAAAAGAACCGGGCGCCCCTGC
GCTGACAGCCGGAACACGGCGGCATCAGAGCAGCCGATTGTCTGTTGTGCCCAGTCATAGCCGAATAGCC
TCTCCACCCAAGCGGCCGGAGAACCTGCGTGCAATCCATCTTGTTCAATCATGCGAAACGATCCTCATCC
TGTCTCTTGATCAGATCATGATCCCCTGCGCCATCAGATCCTTGGCGGCAAGAAAGCCATCCAGTTTACT
TTGCAGGGCTTCCCAACCTTACCAGAGGGCGCCCCAGCTGGCAATTCCGACGTCTTAAGACCCACTTTCA
CATTTAAGTTGTTTTTCTAATCCGCATATAATCAATTCAAGGCCGAATAAGAAGGCTGGCTCTGCACCTT
GGTGATCAAATAATTCGATAGCTTGTCGTAATAATGGCGGCATACTATCAGTAGTAGGTGTTTCCCTTTC
TTCTTTAGCGACTTGATGCTCTTGATCTTCCAATACGCAACCTAAAGTAAAATGCCCCACAGCGCTGAGT
GCATATAATGCATTCTCTAGTGAAAAACCTTGTTGGCATAAAAAGGCTAATTGATTTTCGAGAGTTTCAT
ACTGTTTTTCTGTAGGCCGTGTACCTAAATGTACTTTTGCTCCATCGCGATGACTTAGTAAAGCACATCT
AAAACTTTTAGCGTTATTACGTAAAAAATCTTGCCAGCTTTCCCCTTCTAAAGGGCAAAAGTGAGTATGG
TGCCTATCTAACATCTCAATGGCTAAGGCGTCGAGCAAAGCCCGCTTATTTTTTACATGCCAATACAATG
TAGGCTGCTCTACACCTAGCTTCTGGGCGAGTTTACGGGTTGTTAAACCTTCGATTCCGACCTCATTAAG
CAGCTCTAATGCGCTGTTAATCACTTTACTTTTATCTAATCTAGACATCATTAATTCCTAATTTTTGTTG
ACACTCTATCGTTGATAGAGTTATTTTACCACTCCCTATCAGTGATAGAGAAAAGAATTCAAAAGATCTT
TTAAGAAGGAGATATACAT**ATGGGCGAGTTGGCTCACATTGTTATGGTTGACGCATATAAGCCGACAAAG**
GGAAGTATGCGTAAAGGCGAAGAGCTGTTCACTGGTGTCGTCCCTATTCTGGTGGAACTGGATGGTGATG
TCAACGGTCATAAGTTTTCCGTGCGTGGCGAGGGTGAAGGTGACGCAACTAATGGTAAACTGACGCTGAA
GTTCATCTGTACTACTGGTAAACTGCCGGTACCTTGGCCGACTCTGGTAACGACGCTGACTTATGGTGTT
CAGTGCTTTGCTCGTTATCCGGACCATATGAAGCAGCATGACTTCTTCAAGTCCGCCATGCCGGAAGGCT
ATGTGCAGGAACGCACGATTTCCTTTAAGGATGACGGCACGTACAAAACGCGTGCGGAAGTGAAATTTGA
AGGCGATACCCTGGTAAACCGCATTGAGCTGAAAGGCATTGACTTTAAAGAAGACGGCAATATCCTGGGC
CATAAGCTGGAATACAATTTTAACAGCCACAATGTTTACATCACCGCCGATAAACAAAAAAATGGCATTA
AAGCGAATTTTAAAATTCGCCACAACGTGGAGGATGGCAGCGTGCAGCTGGCTGATCACTACCAGCAAAA
CACTCCAATCGGTGATGGTCCTGTTCTGCTGCCAGACAATCACTATCTGAGCACGCAAAGCGTTCTGTCT
AAAGATCCGAACGAGAAACGCGATCATATGGTTCTGCTGGAGTTCGTAACCGCAGCGGGCATCACGCATG
GTATGGATGAACTGTACAAACTCGAGCACCACCACCACCACCACTGAGGATCCAAACTCGAGTAAGTCGA
GTAAGGATCTCCAGGCATCAAATAAAACGAAAGGCTCAGTCGAAAGACTGGGCCTTTCGTTTTATCTGTT
GTTTGTCGGTGAACGCTCTCTACTAGAGTCACACTGGCTCACCTTCGGGTGGGCCTTTCTGCGTTTATAC
CTAGGGATATATTCCGCTTCCTCGCTCACTGACTCGCTACGCTCGGTCGTTCGACTGCGGCGAGCGGAAA
TGGCTTACGAACGGGGCGGAGATTTCCTGGAAGATGCCAGGAAGATACTTAACAGGGAAGTGAGAGGGCC
GCGGCAAAGCCGTTTTTCCATAGGCTCCGCCCCCCTGACAAGCATCACGAAATCTGACGCTCAAATCAGT
GGTGGCGAAACCCGACAGGACTATAAAGATACCAGGCGTTTCCCCCTGGCGGCTCCCTCGTGCGCTCTCC
TGTTCCTGCCTTTCGGTTTACCGGTGTCATTCCGCTGTTATGGCCGCGTTTGTCTCATTCCACGCCTGAC
ACTCAGTTCCGGGTAGGCAGTTCGCTCCAAGCTGGACTGTATGCACGAACCCCCCGTTCAGTCCGACCGC
TGCGCCTTATCCGGTAACTATCGTCTTGAGTCCAACCCGGAAAGACATGCAAAAGCACCACTGGCAGCAG
CCACTGGTAATTGATTTAGAGGAGTTAGTCTTGAAGTCATGCGCCGGTTAAGGCTAAACTGAAAGGACAA
GTTTTGGTGACTGCGCTCCTCCAAGCCAGTTACCTCGGTTCAAAGAGTTGGTAGCTCAGAGAACCTTCGA
AAAACCGCCCTGCAAGGCGGTTTTTTCGTTTTCAGAGCAAGAGATTACGCGCAGACCAAAACGATCTCAA

GAAGATCATCTTATTAATCAGATAAAATATTTCTAGATTTCAGTGCAATTTATCTCTTCAAATGTAGCAC
CTGAAGTCAGCCCCATACGATATAAGTTGTTACTAGTGCTTGGATTCTCACCAATAAAAAACGCCCGGCG
GCAACCGAGCGTTCTGAACAAATCCAGATGGAGTTCTGAGGTCATTACTGGATCTATCAACAGGAGTCCA
AGCGAGCTCTCGAACCCCAGAGTCCCGCTCAGAAGAACTCGTCAAGAAGGCGATAGAAGGCGATGCGCTG
CGAATCGGGAGCGGCGATACCGTAAAGCACGAGGAAGCGGTCAGCCCATTCGCCGCCAAGCTCTTCAGCA
ATATCACGGGTAGCCAACGCTATGTCCTGATAGCGGTCCGCCACACCCAGCCGGCCACAGTCGATGAATC
CAGAAAAGCGGCCATTTTCCACCATGATATTCGGCAAGCAGGCATCGCCATGGGTCACGACGAGATCCTC
GCCGTCGGGCATGCGCGCCTTGAGCCTGGCGAACAGTTCGGCTGGCGCGAGCCCCTGATGCTCTTCGTCC
A

Supplementary Sequence S5. DNA sequence for the pBbE2K-ΔNterm Ie-ISPS-SpyCatcher

ccgaataagaaggctggctctgcaccttggtgatcaaataattcgatagcttgtcgtaataatggcggca
tactatcagtagtaggtgtttccctttcttctttagcgacttgatgctcttgatcttccaatacgcaacc
taaagtaaaatgccccacagcgctgagtgcatataatgcattctctagtgaaaaaccttgttggcataaa
aaggctaattgattttcgagagtttcatactgtttttctgtaggccgtgtacctaaatgtacttttgctc
catcgcgatgacttagtaaagcacatctaaaacttttagcgttattacgtaaaaaatcttgccagctttc
cccttctaaagggcaaaagtgagtatggtgcctatctaacatctcaatggctaaggcgtcgagcaaagcc
cgcttattttttacatgccaatacaatgtaggctgctctacacctagcttctgggcgagtttacgggttg
ttaaaccttcgattccgacctcattaagcagctctaatgcgctgttaatcactttacttttatctaatct
agacatcattaattcctaatttttgttgacactctatcgttgatagagttattttaccactccctatcag
tgatagagaaaagaattcaaaagatcttttaagaaggagatatacatATGGATAGTGCTACCCATATTAA
ATTCTCAAAACGTGATGAGGACGGCAAAGAGTTAGCTGGTGCAACTATGGAGTTGCGTGATTCATCTGGT
AAAACTATTAGTACATGGATTTCAGATGGACAAGTGAAAGATTTCTACCTGTATCCAGGAAAATATACAT
TTGTCGAAACCGCAGCACCAGACGGTTATGAGGTAGCAACTGCTATTACCTTTACAGTTAATGAGCAAGG
TCAGGTTACTGTAAATGGAGGTAGCGGAGGAAGTGAAAAGGCAGAGTTTTTGACATTATTGGAGCTTATA
GATAATGTCCAGCGCTTGGGATTGGGTTACCGGTTCGAAAGCGACATCCGGGGGGCCTTGGATAGATTTG
TGTCCAGCGGAGGTTTCGATGCGGTGACGAAGACTAGCTTGCACGGCACCGCATTGTCTTTCCGTTTATT
GCGTCAGCACGGGTTCGAAGTCTCACAGGAAGCCTTTTCTGGTTTTAAGGATCAGAATGGGAATTTCCTT
GAGAACCTTAAAGAGGACATCAAGGCAATTTTAAGTTTGTATGAAGCTTCGTTTTTAGCACTGGAGGGCG
AAAACATATTGGACGAAGCTAAGGTGTTCGCTATCTCGCATCTGAAGGAGCTTTCAGAAGAAAAGATCGG
CAAGGAATTAGCCGAGCAGGTGAATCACGCGCTTGAGCTGCCCCTTCATCGCAGAACGCAACGTCTTGAA
GCGGTATGGAGTATCGAGGCGTATCGTAAAAAAGAGGACGCTAACCAAGTTTTATTAGAATTAGCGATCT
TGGACTATAATATGATTCAGAGTGTGTATCAAAGAGACCTTCGGGAGACTTCACGCTGGTGGCGGAGAGT
AGGCTTGGCTACTAAGCTTCATTTCGCACGCGATCGGTTAATAGAGTCGTTTTACTGGGCGGTTGGTGTT
GCATTCGAACCCCAATATTCGGATTGTAGAAATTCCGTAGCCAAAATGTTCAGCTTCGTAACGATTATAG
ATGACATCTATGATGTCTACGGTACTCTGGACGAGCTGGAATTGTTTACGGATGCAGTTGAGCGCTGGGA
TGTCAACGCTATTAACGATCTGCCTGACTACATGAAGTTATGCTTTCTTGCCCTTTACAACACCATCAAT
GAGATAGCTTACGACAACCTGAAAGATAAGGGAGAGAACATATTACCATATTTGACAAAGGCCTGGGCAG
ACTTATGCAATGCGTTTCTTCAGGAGGCAAAGTGGCTTTACAACAAAAGTACGCCAACGTTCGACGACTA
CTTCGGTAATGCGTGGAAGTCCAGTTCTGGACCCCTTCAGTTAGTGTTCGCGTACTTCGCGGTAGTCCAA
AACATCAAGAAGGAGGAGATCGAAAATTTACAAAAGTACCATGACACGATTTCACGTCCCAGCCACATTT
TTCGTTTATGCAATGATCTGGCCTCCGCCTCTGCAGAAATAGCACGGGGAGAGACCGCTAATTCAGTATC
CTGCTACATGCGGACAAAGGGGATATCGGAAGAACTTGCGACAGAAAGCGTTATGAACCTGATCGACGAG
ACCTGGAAGAAAATGAACAAAGAAAAGTTAGGAGGTTCCCTTTTTGCAAAGCCATTTGTTGAAACTGCGA
TTAATTTGGCAAGACAGTCACACTGTACCTACCACAACGGAGATGCTCACACGAGCCCAGATGAGCTTAC
ACGCAAGAGAGTTCTTTCGGTCATCACAGAACCTATTCTGCCATTTGAGCGTTAAggatccaaactcgag
taaggatctccaggcatcaaataaaacgaaaggctcagtcgaaagactgggcctttcgttttatctgttg
tttgtcggtgaacgctctctactagagtcacactggctcaccttcgggtgggcctttctgcgtttatacc
tagggcgttcggctgcggcgagcggtatcagctcactcaaaggcggtaatacggttatccacagaatcag
gggataacgcaggaaagaacatgtgagcaaaaggccagcaaaaggccaggaaccgtaaaaaggccgcgtt
gctggcgtttttccataggctccgcccccctgacgagcatcacaaaaatcgacgctcaagtcagaggtgg
cgaaacccgacaggactataaagataccaggcgtttccccctggaagctccctcgtgcgctctcctgttc
cgaccctgccgcttaccggatacctgtccgcctttctcccttcgggaagcgtggcgctttctcatagctc
acgctgtaggtatctcagttcggtgtaggtcgttcgctccaagctgggctgtgtgcacgaaccccccgtt
cagcccgaccgctgcgccttatccggtaactatcgtcttgagtccaacccggtaagacacgacttatcgc

cactggcagcagccactggtaacaggattagcagagcgaggtatgtaggcggtgctacagagttcttgaa
gtggtggcctaactacggctacactagaaggacagtatttggtatctgcgctctgctgaagccagttacc
ttcggaaaaagagttggtagctcttgatccggcaaacaaaccaccgctggtagcggtggtttttttgttt
gcaagcagcagattacgcgcagaaaaaaaggatctcaagaagatcctttgatcttttctacggggtctga
cgctcagtggaacgaaaactcacgttaagggattttggtcatgactagtgcttggattctcaccaataaa
aaacgcccggcggcaaccgagcgttctgaacaaatccagatggagttctgaggtcattactggatctatc
aacaggagtccaagcgagctctcgaaccccagagtcccgctcagaagaactcgtcaagaaggcgatagaa
ggcgatgcgctgcgaatcgggagcggcgataccgtaaagcacgaggaagcggtcagcccattcgccgcca
agctcttcagcaatatcacgggtagccaacgctatgtcctgatagcggtccgccacacccagccggccac
agtcgatgaatccagaaaagcggccattttccaccatgatattcggcaagcaggcatcgccatgggtcac
gacgagatcctcgccgtcgggcatgcgcgccttgagcctggcgaacagttcggctggcgcgagcccctga
tgctcttcgtccagatcatcctgatcgacaagaccggcttccatccgagtacgtgctcgctcgatgcgat
gtttcgcttggtggtcgaatgggcaggtagccggatcaagcgtatgcagccgccgcattgcatcagccat
gatggatactttctcggcaggagcaaggtgagatgacaggagatcctgccccggcacttcgcccaatagc
agccagtcccttcccgcttcagtgacaacgtcgagcacagctgcgcaaggaacgcccgtcgtggccagcc
acgatagccgcgctgcctcgtcctgcagttcattcagggcaccggacaggtcggtcttgacaaaaagaac
cgggcgcccctgcgctgacagccggaacacggcggcatcagagcagccgattgtctgttgtgcccagtca
tagccgaatagcctctccacccaagcggccggagaacctgcgtgcaatccatcttgttcaatcatgcgaa
acgatcctcatcctgtctcttgatcagatcatgatcccctgcgccatcagatccttggcggcaagaaagc
catccagtttactttgcagggcttcccaaccttaccagagggcgccccagctggcaattccgacgtctta
agacccactttcacatttaagttgtttttctaatccgcatataatcaattcaagg
